# JWA binding to NCOA4 alleviates degeneration in dopaminergic neurons through suppression of ferritinophagy in Parkinson's disease

**DOI:** 10.1016/j.redox.2024.103190

**Published:** 2024-05-13

**Authors:** Xinxin Zhao, Zhengwei Kang, Ruixue Han, Min Wang, Yueping Wang, Xin Sun, Cong Wang, Jianwei Zhou, Lei Cao, Ming Lu

**Affiliations:** a Jiangsu Key Laboratory of Neurodegeneration, Department of Pharmacology, Nanjing Medical University, Nanjing 211166, China; b Department of Molecular Cell Biology & Toxicology, Center for Global Health, School of Public Health, Nanjing Medical University, 211166, Nanjing, China; c Changzhou Second People's Hospital, Changzhou Medical Center, Nanjing Medical University, 213000, Changzhou, China

**Keywords:** JWA, Ferroptosis, Ferritinophagy, JAC4, Neuroprotection, Parkinson's disease

## Abstract

Parkinson's disease (PD) poses a significant challenge in neurodegenerative disorders, characterized by the progressive loss of dopaminergic (DA) neurons in the substantia nigra pars compacta (SNc). The intricate mechanisms orchestrating DA neurodegeneration in PD are not fully understood, necessitating the exploration of innovative therapeutic approaches. Recent studies have implicated ferroptosis as a major contributor to the loss of DA neurons, revealing a complex interplay between iron accumulation and neurodegeneration. However, the sophisticated nature of this process challenges the conventional belief that mere iron removal could effectively prevent DA neuronal ferroptosis. Here, we report JWA, alternatively referred to as ARL6IP5, as a negative regulator of ferroptosis, capable of ameliorating DA neuronal loss in the context of PD. In this study, synchronized expression patterns of JWA and tyrosine hydroxylase (TH) in PD patients and mice were observed, underscoring the importance of JWA for DA neuronal survival. Screening of ferroptosis-related genes unraveled the engagement of iron metabolism in the JWA-dependent inhibition of DA neuronal ferroptosis. Genetic manipulation of *JWA* provided compelling evidence linking its neuroprotective effects to the attenuation of NCOA4-mediated ferritinophagy. Molecular docking, co-immunoprecipitation, and immunofluorescence studies confirmed that JWA mitigated DA neuronal ferroptosis by occupying the ferritin binding site of NCOA4. Moreover, the JWA-activating compound, JAC4, demonstrated promising neuroprotective effects in cellular and animal PD models by elevating JWA expression, offering a potential avenue for neuroprotection in PD. Collectively, our work establishes JWA as a novel regulator of ferritinophagy, presenting a promising therapeutic target for addressing DA neuronal ferroptosis in PD.

## Graphical abstract

**Figure:**
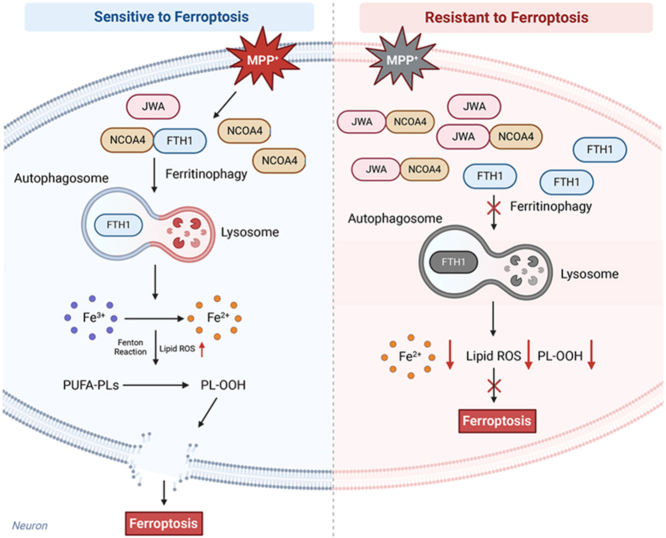

## 1 Introduction

Parkinson's disease (PD) stands as a formidable challenge in the realm of neurodegenerative disorders, characterized by the progressive loss of dopaminergic (DA) neurons in the substantia nigra pars compacta (SNc) [1]. The current therapeutic landscape for PD primarily revolves around symptomatic management, with levodopa serving as a gold standard [2]. However, the absence of a curative strategy underscores the urgent need for neuroprotective interventions capable of impeding or decelerating the ongoing degeneration of DA neurons. Despite extensive research efforts [3,4], the precise mechanisms orchestrating DA neurodegeneration in PD remain enigmatic, necessitating a deeper exploration into novel therapeutic avenues.

The discovery of ferroptosis, an iron-dependent form of regulated cell death defined in 2012 [5], has brought new dimensions to understanding the demise of DA neurons in PD. Intriguingly, iron accumulation and lipid peroxidation were observed in PD patients and animal models before the formal recognition of ferroptosis [[6], [7], [8]]. Recent studies have implicated ferroptosis as a major contributor to the loss of DA neurons, revealing the intricate interplay between iron metabolism, lipid peroxidation, and neurodegeneration [4,9,10]. However, the complexity of this process extends beyond a simplistic imbalance in iron levels, challenging the conventional notion that merely removing iron could effectively prevent DA neuronal ferroptosis. A noteworthy intervention in iron dynamics is the use of the iron chelator deferiprone, initially approved for treating transfusion-dependent thalassemia [11]. Deferiprone treatment exhibited a paradoxical impact on PD during a phase 2 randomized clinical trial. Despite its effectiveness in reducing iron content, deferiprone exacerbated parkinsonism [12]. This paradoxical outcome emphasizes the intricate and multifaceted nature of iron dynamics in the context of PD. In light of this, there arises a compelling need to identify novel molecular regulators capable of delicately balancing iron content, offering novel perspectives on potential therapeutic interventions.

Amidst these challenges, our focus shifts to *JWA*, also known as *ARL6IP5*, initially identified as an all-trans retinoic acid-responsive gene [13], playing multifaceted roles in cellular homeostasis, including the modulation of reactive oxygen species (ROS) and glutathione (GSH) and glutathione peroxidase levels [14,15], key indicators of ferroptosis. Moreover, previous work from our group and others has emphasized the indispensable role of JWA in ensuring the survival of DA neurons in PD [[16], [17], [18], [19]]. Nevertheless, the potential involvement of JWA in DA neuronal ferroptosis has remained elusive. Given its apparent relevance to ferroptosis and its well-established role in safeguarding the survival of DA neurons, we postulate that JWA may function as a negative regulator of ferroptosis in DA neurons.

In this study, we aim to unravel the intricate regulatory role of JWA in ferroptosis, particularly concerning DA neuronal degeneration in PD. We explored the expression correlation between JWA and tyrosine hydroxylase (TH) in PD patients and mice. Through the screening of ferroptosis-related genes, we identified that iron metabolism was involved in the JWA-dependent inhibition of DA neuronal ferroptosis. Genetic manipulation of *JWA* provided compelling evidence that the neuroprotection afforded by JWA was associated with the blunting of NCOA4-mediated ferritinophagy. Subsequently, we predicted the interaction between JWA and NCOA4 using molecular docking methods. Validated through Co-IP and immunofluorescence of JWA and NCOA4, we identified that JWA mitigated DA neuronal ferroptosis by occupying the ferritin binding site of NCOA4. Additionally, our study introduces a synthesized JWA-activating compound, JAC4, which amplifies JWA expression and exhibits promising neuroprotective effects in cellular and animal models of PD. By targeting the precise mechanism of ferritinophagy, we envisage that strategies focused on increasing JWA expression, exemplified by JAC4 treatment, could offer a novel avenue for neuroprotection in PD.

## 2 Materials and methods

### 2.1 Animals

C57BL/6 male mice, aged 8–10 weeks and weighing 20–25 g, were procured from the Animal Resource Center of the Faculty of Medicine, Nanjing Medical University. The mice were bred and housed in the Animal Resource Centre of the Faculty of Medicine, Nanjing Medical University, with unrestricted access to food and water. They were maintained under standard laboratory conditions, including an ambient temperature of 22 °C ± 2 °C and a 12/12 h (h)-light/dark cycle. All animal experiments strictly adhered to ethical regulations and received approval from the Institutional Animal Care and Use Committee (IACUC No. 1601153–3) of the Nanjing Medical University Experimental Animal Department.

### 2.2 The MPTP mouse model and JAC4 treatment

The chronic PD model was established in male C57BL/6 mice using 1-methyl-4-phenyl-1,2,3,6-tetrahydropyridine (MPTP), following previously established procedures [20]. In brief, mice received subcutaneous injections of 20 mg/kg MPTP in saline, followed by intraperitoneal injections of 250 mg/kg probenecid in DMSO 1 h later, repeated twice per week over a 5-week period. After a 1-week rest, motor symptom evaluation through behavioral tests was conducted, and samples were collected. JAC4 was provided by our collaborator, Prof Jianwei Zhou [21]. Throughout this period, JAC4 was administered intragastrically at doses of 25, 50, and 100 mg/kg per day. Reagents and resources were listed in the Supplementary Table 1.

### 2.3 Behavioral tests

Behavioral assessments, including the open field test, pole test, and rotarod test, were conducted in accordance with established protocols [22].

The open field test primarily assessed voluntary movement ability. Mice were acclimated to the experimental environment for 3 h before being individually placed into the central area of an opaque box (50 × 50 × 40 cm), divided into a central region (side length: 35 cm) and a border area. Video recording of each mouse lasted for 10 min, and the opaque box was sterilized with alcohol before each test to eliminate residual mouse odor. The TopScan Realtime Option (CleverSys Inc) was employed to analyze the travel paths and total distance of mice.

The pole test, widely used for evaluating basal ganglia-related movement disorders in mice, recorded the time taken for mice to climb from the top to the bottom of a smooth pole (diameter: 1 cm, height: 50 cm), landing on both front claws. Mice with motor coordination defects typically took longer. The mice underwent three training sessions, and the total time spent from the top to the bottom was recorded for each trial.

The rotarod test evaluated the motor coordination and balance of mice. Before testing, mice were trained on an accelerating rotarod rod in a separate compartment. During the test, mice were subjected to a speed of 20 rpm for 180 s, and the latency to fall off the rod was recorded using the JLBehv-RRTG-5 system.

### 2.4 Cell culture and treatment

SH-SY5Y cells (ATCC, CRL-2266) were cultured and differentiated according to established protocols [23]. In brief, cells were cultured in basic growth medium (DMEM: F12 with 10 % FBS, 1 x penicillin/streptomycin) and seeded onto poly-l-ornithine/fibronectin pre-coated 6-well plates at a density of 15 × 10^4^ cells/well. After 24 h, the culture medium was replaced with Stage I medium (DMEM: F12 with 2.5 % FBS, 10 μM retinoic acid, 1 x penicillin/streptomycin), and cells were maintained for 5 days. Subsequently, Stage II medium (DMEM: F12 with 1 % N2 supplement, 50 ng/ml BDNF, 20 mM KCl, 1 x penicillin/streptomycin) was applied, and cells were maintained for an additional 5 days. Cell transfection occurred following the successful differentiation of SH-SY5Y cells into dopamine-like neurons. Differentiated SH-SY5Y cells were pretreated with deferiprone (DFO, 0.1–500 μM, HY-B0568, MedChemExpress) for 1 h, followed by MPP + treatment (500 μM) for 24 h. In Erastin-induced ferroptosis model, Ferrostatin-1 (10 μM, HY-100579, MedChemExpress) was pretreated for 1 h, followed by Erastin treatment (10 μM, HY-15763, MedChemExpress) for 24 h.

Primary neurons were cultured as previously outlined [24]. Briefly, the midbrain was meticulously dissected in ice-cold PBS, dissociated using 0.25 % trypsin (12 min at 37 °C), and plated onto poly-*d*-lysine–treated culture plates in DMEM supplemented with 10 % fetal bovine serum and 1 % penicillin/streptomycin. After a 4-h incubation in a 5 % CO2 incubator at 37 °C, the medium was replaced with serum-free Neurobasal/B27/glutamine medium. Half of the medium was refreshed every 3 days. All experiments were conducted on neurons cultured for 12–14 days in vitro. Neurons underwent pretreatment with JAC4 at concentrations of 0.1, 1, and 10 μM for 1 h, followed by MPP+ (30 μM) treatment for 24 h.

### 2.5 Cell transfection and plasmid construction

For differentiated SH-SY5Y cells cultured in 6-well plates, siRNA targeting *JWA* or negative control (NC) siRNA (Jima, Shanghai, China) was transfected using siRNA-Mate (Jima, Shanghai, China) in OPTI-MEM reduced serum medium (Gibco). Plasmids containing *JWA* (BC005143, PPL, China) were transfected into differentiated SH-SY5Y cells using lipofectamine 3000 reagent (Invitrogen, Carlsbad, CA, USA). Cells were utilized for subsequent experiments 48 h post-transfection. The sequences of siRNA are shown below:

Si-*ARL6IP5* (*JWA*) (Homo sapiens)
*JWA* (sense): CGAGCUAUUUCCUUAUCUCTT
*JWA* (antisense): GAGAUAAGGAAAUAGCUCGTT

### 2.6 Immunofluorescence and immunohistochemistry

Immunofluorescence and immunohistochemistry were performed following the protocols described in our prior study [22]. Cells or brain slices (frozen sections, 30 μm) were fixed in 4 % paraformaldehyde, blocked with 5 % BSA in PBST (0.3 % Triton X-100), and then incubated with desired primary antibodies at 4 °C overnight. The corresponding secondary antibodies were subsequently incubated for 1 h at room temperature. Image J (1.53q) was utilized for colocalization analysis as previously described [25], images for each channel were separately thresholded, and colocalization was defined as at least one pixel of overlap between the two channels. Neurite length was measured using Image J (1.53q). Stereology was performed for cell counting using the optical fractionator (Stereo Investigator 7, MBF Bioscience, Williston, VT). Every sixth coronal frozen section was collected for quantification. The regions of SNpc in the midbrain sections were outlined at low magnification (40×), and the counting frame size was 50 μm × 50 μm, with a sampling grid size of 100 μm × 100 μm. All stereological analyses were conducted under the 200× magnification of an Olympus BX52 microscope (Olympus America Inc., Melville, NY). The primary and secondary antibodies used in this study were listed in the Supplementary Table 1.

### 2.7 Perls Prussian blue staining

Brain slices underwent a thorough washing procedure with PBS prior to Perls Prussian Blue staining. The preparation of the Perls staining and the enhancement working solution adhered to the guidelines outlined in the Perls Prussian blue staining kit (G1428, Solarbio, China). Subsequent to completing the staining protocol as per the provided instructions, the brain slices underwent sequential dehydration in 70 % ethanol for 30 s, 80 % ethanol for 60 s, 90 % ethanol for 60 s, and absolute ethanol for 2 min. Following this, the slices were cleared in xylene for 5 min. Following the application of neutral resin for mounting, the slices underwent observation and microscopic imaging.

### 2.8 Live cell staining

Live cell staining was conducted in SH-SY5Y cells and primary neurons. FerroOrange staining (F374, DOJINDO, Japan) at a concentration of 1 μM was employed for observing ferrous ions. C11 BODIPY 581/591 (D3861, Invitrogen) was used at 5 μM to visualize lipid peroxidation. LysoTracker Deep Red (L12492, Invitrogen) at 50 nM was utilized to observe lysosomes. All staining procedures strictly followed the manufacturer's instructions. Nuclei were stained using Hoechst 33,342 (H1399, Invitrogen). Fluorescent microscopy was utilized for image acquisition, and Image J (1.53q) was employed for quantifying fluorescence intensity. A uniform color threshold was applied across all images, and corrected fluorescence was determined by subtracting background signals using the “analyze particles” function.

### 2.9 Cell viability assay

Cell viability was evaluated utilizing a commercial CCK-8 kit (Selleck, Houston, TX, USA) following the manufacturer's guidelines. Briefly, SH-SY5Y cells and primary neurons were seeded at a density of 5000 cells/well in a 96-well plate. After the designated treatments, cells were exposed to the CCK-8 reagent (10 μl/well) for 1 h. Subsequently, the plate was gently agitated on an orbital shaker for 1 min, and the absorbance at 450 nm was quantified using the Multiskan Spectrum (Thermo Fisher Scientific).

### 2.10 LDH measurement

The assessment of LDH release was conducted concurrently with the cell viability assay. Briefly, the supernatant obtained from SH-SY5Y cells and primary neurons during the cell viability assay was collected. The LDH content in the supernatant was determined following the manufacturer's instructions (BC0685, Solarbio, China).

### 2.11 GSH/GSSG, MDA, and iron measurement

The quantification of GSH/GSSG, MDA, and iron content was conducted using commercial kits. In brief, the levels of glutathione (GSH) and oxidized glutathione (GSSG) were determined according to the manufacturer's instructions (S0053, Beyotime, China). The assessment of malondialdehyde (MDA) levels was performed using the commercial kit (A003-4-1, Nanjing Jiancheng Bioengineering Institute, China). The measurement of iron content was carried out following the instructions provided in the commercial kit (A039-2-1, Nanjing Jiancheng Bioengineering Institute, China).

### 2.12 Real time quantitative and reverse transcription PCR (RT-qPCR)

Total RNA extraction from SH-SY5Y cells was carried out using trizol reagent (Invitrogen, Carlsbad, CA, USA). Reverse transcription of the total RNA was conducted using 5 × HiscriptⅡ qRT supermix (Vazyme, Nanjing, China). Real-time PCR was performed using SYBR Green Master Mix (Vazyme, Nanjing, China) in a StepOnePlus instrument (Applied Biosystems). The relative quantity was calculated using the −ΔΔCT and 2^−ΔΔCT^ methods. The sequences of primers (Generay, Shanghai, China) utilized for qPCR were listed in the Supplementary Table 2.

### 2.13 Western blotting analysis and Co-immunoprecipitation (CO-IP) assay

Cells and brain tissues underwent lysis in RIPA buffer (Bio-Rad), which was supplemented with a protease phosphatase inhibitor cocktail (Roche, USA). Following centrifugation at 13,000 rpm for 20 min at 4 °C, the protein concentration in the lysates was determined using the Micro BCA Kit (Beyotime, Shanghai, China). Subsequently, proteins were separated through SDS-PAGE on an 8–12 % gradient separating gel and transferred onto PVDF membranes. After blocking, the membranes were incubated overnight at 4 °C with specific primary antibodies. After three washes, corresponding HRP-conjugated secondary antibodies were applied for 1 h at room temperature. Protein detection utilized Pierce detection reagents (Thermo Fisher Scientific), and analysis of protein bands was carried out using the ImageQuant™ LAS 4000 imaging system (GE Healthcare, Pittsburgh, PA, USA).

For the Co-IP assay, cell lysis occurred in NP-40 buffer (Thermo Fisher Scientific), and proteins were immunoprecipitated using specific primary antibodies (1 μg antibody per 100 μg of protein) and MagnaBindTM Protein G Magnetic Beads (Thermo Fisher Scientific, Waltham, MA, USA). Post-immunoprecipitation, proteins were separated from the beads following the manufacturer's instructions. The prepared samples underwent western blotting after heating at 95 °C for 10 min. Details of the primary and secondary antibodies utilized in this study can be found in Supplementary Table 1.

### 2.14 Simulation of the interaction between JWA and NCOA4

The three-dimensional structures of JWA (AlphaFold ID: O75915) and NCOA4 (AlphaFold ID: AF-Q13772-F1) were acquired from the AlphaFold Protein Structure Database (https://alphafold.ebi.ac.uk/). To investigate the interaction between JWA and NCOA4, we employed the ZDOCK module of Discovery Studio 2019. In this analysis, JWA was designated as the ligand, while NCOA4 served as the receptor, utilizing a final angular step size of 6 for conformational sampling. The selection of the final docked conformations from each docking process was based on the best ZDOCK and RDOCK scores. The resulting visual representations were generated using Pymol version Open-Source 1.6. x.

### 2.15 High-performance liquid chromatography (HPLC) analysis

Neurotransmitter levels in the striatal samples were assessed using high-performance liquid chromatography (HPLC), following previously established protocols [26]. The samples underwent homogenization in 0.1 M perchloric acid (1 mg tissue in 100 μL perchloric acid), ultrasonication, and subsequent centrifugation at 20,000 rpm for 30 min at 4 °C. The resulting supernatant was collected for the quantification of dopamine and its metabolites, along with 5-HT and its metabolite. Detection was carried out using the HPLC-EDC system from Thermo Fisher Scientific, USA. The mobile phase comprised a mixed solution of OSA (1.7 mM), NaH_2_PO_4_·2H_2_O (90.0 mM), C_6_H_8_O_7_·H_2_O (50.0 mM), EDTA·2Na (50.0 μM), and 5 % acetonitrile, with a flow rate of 0.2 ml/min. Monitoring was performed by an electrochemical detector set at 350 mV.

### 2.16 Statistical analysis

Data analysis was carried out using GraphPad Prism 8.0.0 software. A single-variable comparison between two groups was performed using an unpaired Student's *t*-test. For comparisons involving one variable within three or more groups, one-way analysis of variance (ANOVA) was applied, and post-hoc analysis was conducted using Tukey's multiple comparisons test. In instances involving comparisons between two independent variables, two-way ANOVA was employed, followed by Tukey's multiple comparisons test. Graphs were generated to illustrate Mean ± SEM, and individual data points were included. Statistical significance was considered at p < 0.05. The in vitro experiments were conducted with a minimum of three biological replicates and two technical replicates, and additional details can be found in the figure legends.

## 3 Results

### 3.1 JWA and TH exhibit synchronized expression patterns in PD patients and mice

**Fig. 1:**
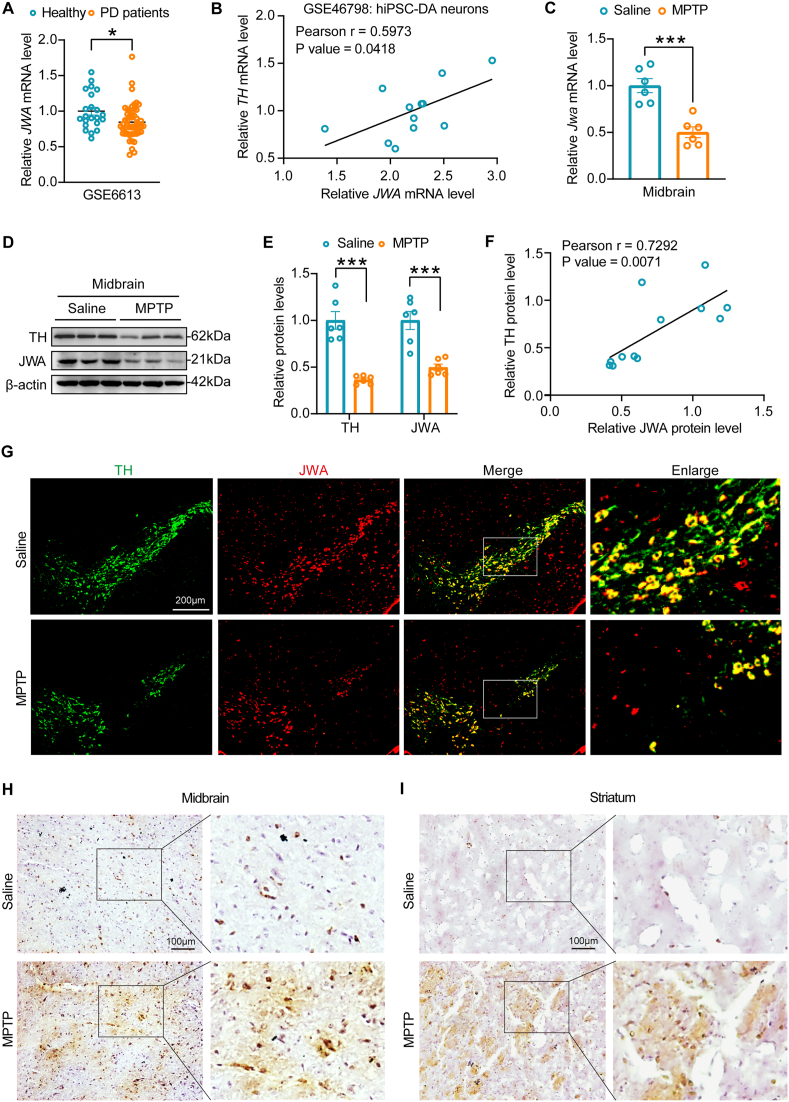
Synchronized expression patterns of JWA and TH in PD patients and mice. **(A)** Assessment of *JWA* mRNA levels in healthy individuals and PD patients using GEO dataset GSE6613. *p < 0.05 determined by unpaired *t*-test, n = 22 in the healthy group and n = 50 in the PD group. **(B)** Examination of the correlation between *JWA* and *TH* mRNA levels in human induced pluripotent stem cell (iPSC)-derived dopaminergic (DA) neurons from PD patients in GSE46798 dataset. A Pearson correlation coefficient test revealed a statistically significant linear correlation (r = 0.5973, p = 0.0418, n = 12). **(C)** RT-qPCR analysis of *Jwa* mRNA levels in the midbrain of mice exposed to Saline and MPTP. ***p < 0.001 by unpaired *t*-test, n = 6. **(D**–**E)** Western blotting analysis and quantification of TH and JWA expression in the midbrain of mice exposed to Saline and MPTP. ***p < 0.001 by unpaired *t*-test, n = 6. **(F)** Evaluation of the linear correlation between TH and JWA expression in the midbrain of mice exposed to Saline and MPTP. Pearson r = 0.7292, p = 0.0071, determined by Pearson correlation coefficient test, n = 12. **(G)** Immunofluorescence staining of TH and JWA in the SNc region of mice exposed to Saline and MPTP. Scale bar: 200 μm. **(H–I)** Perls Prussian blue staining of brain slices in the midbrain and striatum of mice subjected to Saline and MPTP. Scale bar: 100 μm. (For interpretation of the references to color in this figure legend, the reader is referred to the Web version of this article.)

To investigate the role of JWA in PD, we analyzed the GEO dataset GSE6613 and found a noteworthy decline in *JWA* mRNA level in PD patients (Fig. 1A). Intriguingly, a positive correlation between *JWA* mRNA level and tyrosine hydroxylase (*TH*) mRNA level was observed in human induced pluripotent stem cell (iPSC)-derived dopaminergic (DA) neurons from PD patients, as indicated by the analysis of GSE46798 (Fig. 1B). These clinical findings underscore the potential significance of JWA in the survival of DA neurons in PD. To substantiate this observation, we utilized a 1-methyl-4-phenyl-1,2,3,6-tetrahydropyridine (MPTP)-induced mouse PD model. Concordantly, a significant reduction in *Jwa* mRNA level was observed in the midbrain of PD mice (Fig. 1C). Moreover, both TH and JWA protein levels were decreased in the midbrain of PD mice (Fig. 1D–E). Consistent with the GSE46798 results, the protein level of JWA exhibited a positive correlation with TH in PD mice (Fig. 1F). Immunostaining of TH and JWA in the substantia nigra pars compacta (SNc) region of the midbrain revealed a substantial loss of DA neurons accompanied by a reduced expression of JWA in PD mice (Fig. 1G). Intriguingly, Perls Prussian blue staining of midbrain and striatum slices identified a significant accumulation of iron in MPTP-induced PD mice (Fig. 1H–I). Taken together, our findings unveil a synchronized expression pattern of JWA and TH in PD, suggesting a potential neuroprotective role of JWA in the context of DA neuronal loss, possibly linked to iron accumulation.

### 3.2 JWA protects against ferroptosis of SH-SY5Y cells in the MPP + model

Building upon reported functions of JWA in diminishing ROS production and enhancing glutathione and glutathione peroxidase levels [14,15], pivotal indicators of ferroptosis, we delved into investigating the role of JWA in the context of DA neuronal ferroptosis associated with PD. In the MPTP-induced PD model, a diminished ratio of GSH/GSSG and elevated production of MDA in the midbrain of PD mice were observed, indicating reduced antioxidative capacity and heightened lipid peroxidation in the PD context (Figs. S1A–B). Additionally, there was noticeable accumulation of iron in the midbrain of PD mice (Fig. S1C). Remarkably, these trends were consistently observed in the striatum of PD mice as well (Figs. S1D–F). Collectively, these results provide compelling evidence for the occurrence of ferroptosis in the nigrostriatal pathway within the context of PD. In the MPP + model, we observed a partial restoration of SH-SY5Y cell viability with deferoxamine (DFO) treatment (Fig. S1G), an iron chelator clinically approved for use [27]. Furthermore, DFO treatment mitigated the elevation of MDA and iron levels induced by MPP+ in SH-SY5Y cells (Figs. S1H–I). This substantiates the occurrence of ferroptosis in the progression of PD and underscores the potential of inhibiting ferroptosis as a promising strategy to prevent the loss of DA neurons.

**Fig. 2:**
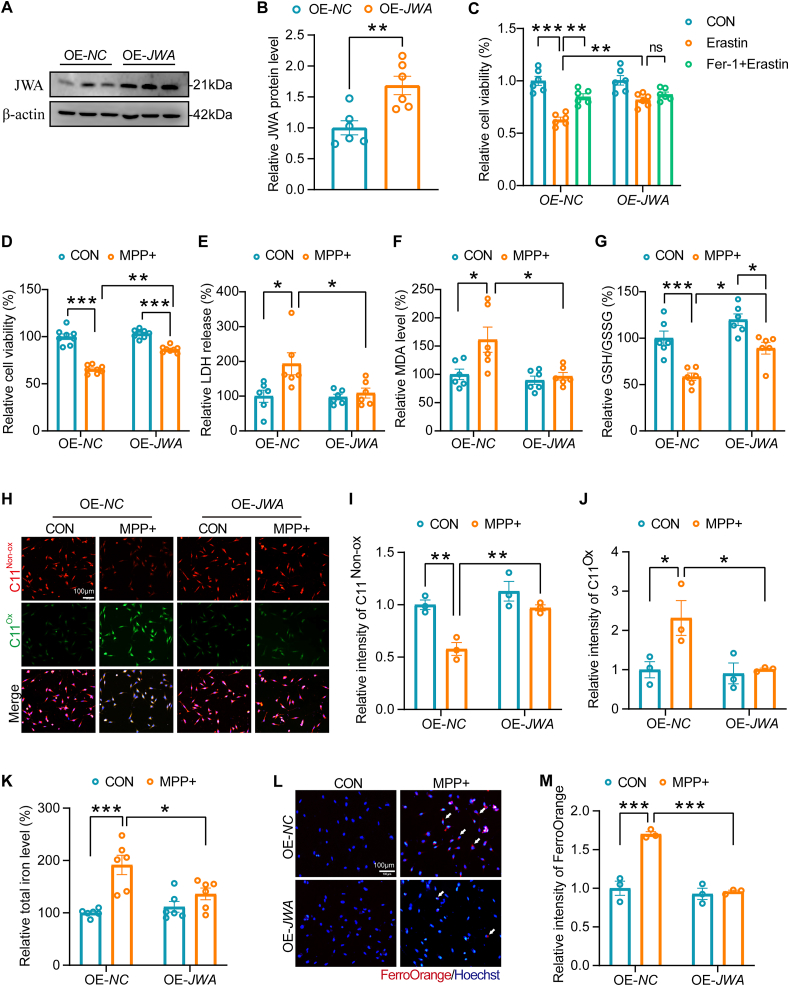
*JWA* overexpression improves the resistance of MPP + -treated SH-SY5Y cells to ferroptosis. **(A**–**B)** Evaluation of *JWA* overexpression efficiency in SH-SY5Y cells through Western blotting analysis and quantification. **p < 0.01 by unpaired *t*-test, n = 6. **(C)** Effect of *JWA* overexpression on Erastin-induced ferroptosis model. **p < 0.01, ***p < 0.001 by two-way ANOVA with Tukey's multiple comparisons test, n = 6, ns: no significance. **(D)** Assessment of cell viability via CCK-8 assay in WT and *JWA*-overexpressing SH-SY5Y cells exposed to MPP + or untreated. **p < 0.01, ***p < 0.001 by two-way ANOVA with Tukey's multiple comparisons test, n = 8. **(E)** Measurement of LDH release in WT and *JWA*-overexpressing SH-SY5Y cells exposed to MPP + or untreated. *p < 0.05 by two-way ANOVA with Tukey's multiple comparisons test, n = 6. **(F)** Quantification of MDA levels in WT and *JWA*-overexpressing SH-SY5Y cells exposed to MPP + or untreated. *p < 0.05 by two-way ANOVA with Tukey's multiple comparisons test, n = 6. **(G)** Assessment of the GSH/GSSG ratio in WT and *JWA*-overexpressing SH-SY5Y cells exposed to MPP + or untreated. *p < 0.05, ***p < 0.001 by two-way ANOVA with Tukey's multiple comparisons test, n = 6. **(H**–**J)** Staining and quantification of the reduced and oxidized forms of C11-BODIPY in WT and *JWA*-overexpressing SH-SY5Y cells exposed to MPP + or untreated. *p < 0.05, **p < 0.01 by two-way ANOVA with Tukey's multiple comparisons test, n = 3, scale bar: 100 μm. **(K)** Measurement of total iron levels in WT and *JWA*-overexpressing SH-SY5Y cells exposed to MPP + or untreated. *p < 0.05, ***p < 0.001 by two-way ANOVA with Tukey's multiple comparisons test, n = 6. **(L**–**M)** FerroOrange staining and quantification of ferrous ion in WT and *JWA*-overexpressing SH-SY5Y cells exposed to MPP + or untreated. ***p < 0.001 by two-way ANOVA with Tukey's multiple comparisons test, n = 3, scale bar: 100 μm.

Next, we aimed to uncover the regulatory role of JWA in ferroptosis of SH-SY5Y cells treated with MPP+. Overexpression of *JWA* was performed in SH-SY5Y cells (Fig. 2A–B). Intriguingly, overexpression of *JWA* rescued the loss of TH expression in MPP + -treated differentiated SH-SY5Y cells, but it did not impact TH expression under normal condition (Fig. S1J-L). This suggests that JWA advocates for the survival of dopaminergic neurons and indirectly boosts the protein level of TH instead of stimulating its expression. To elucidate JWA's role in ferroptosis, we conducted an Erastin-induced ferroptosis model using SH-SY5Y cells with *JWA* overexpression. Our findings demonstrated that *JWA* overexpression inhibits Erastin-induced ferroptosis, mirroring the effect of Ferrostatin-1 treatment (Fig. 2C). Notably, *JWA* overexpression mitigated the reduced SH-SY5Y cell viability and inhibited LDH release in the context of MPP + treatment (Fig. 2D–E). Furthermore, *JWA* overexpression restored the increased MDA level and reduced ratio of GSH/GSSG induced by MPP + treatment in SH-SY5Y cells (Fig. 2F–G). Subsequently, we employed the C11-BODIPY probe to assess lipid peroxidation in SH-SY5Y cells. Notably, MPP + treatment induced substantial oxidation of C11-BODIPY accompanied by a decrease in the reduced form of C11-BODIPY. In contrast, the overexpression of *JWA* resulted in an increase in the reduced form and a decrease in the oxidized form of C11-BODIPY, indicative of the inhibition of lipid peroxidation in the MPP + model (Fig. 2H–J). Importantly, *JWA* overexpression reduced the total iron level and Fe^2+^ ions in SH-SY5Y cells with MPP + treatment, as visualized by FerroOrange staining (Fig. 2K-M). To validate the inhibitory effect of JWA on ferroptosis, *JWA* knockdown SH-SY5Y cells were generated (Figs. S2A–B). *JWA* knockdown intensified Erastin-induced ferroptosis, a situation that was alleviated with Ferrostatin-1 treatment (Fig. S2C). These cells exhibited increased susceptibility to MPP + -induced injury, as evidenced by exacerbated reduction in cell viability and increased LDH release (Figs. S2D–E). Consistent with the findings in SH-SY5Y cells with *JWA* overexpression, *JWA* knockdown increased lipid peroxidation and reduced antioxidative capacity in SH-SY5Y cells with MPP + treatment (Figs. S2F–J). Moreover, a substantial amount of total iron and Fe^2+^ ions were observed in SH-SY5Y cells with *JWA* knockdown in the MPP + model (Fig. S2K-M). These results collectively reveal that JWA serves as a protective factor against ferroptosis in SH-SY5Y cells within the MPP + model.

### 3.3 JWA impedes ferroptosis of SH-SY5Y cells through inhibiting ferritinophagy in the MPP + model

**Fig. 3:**
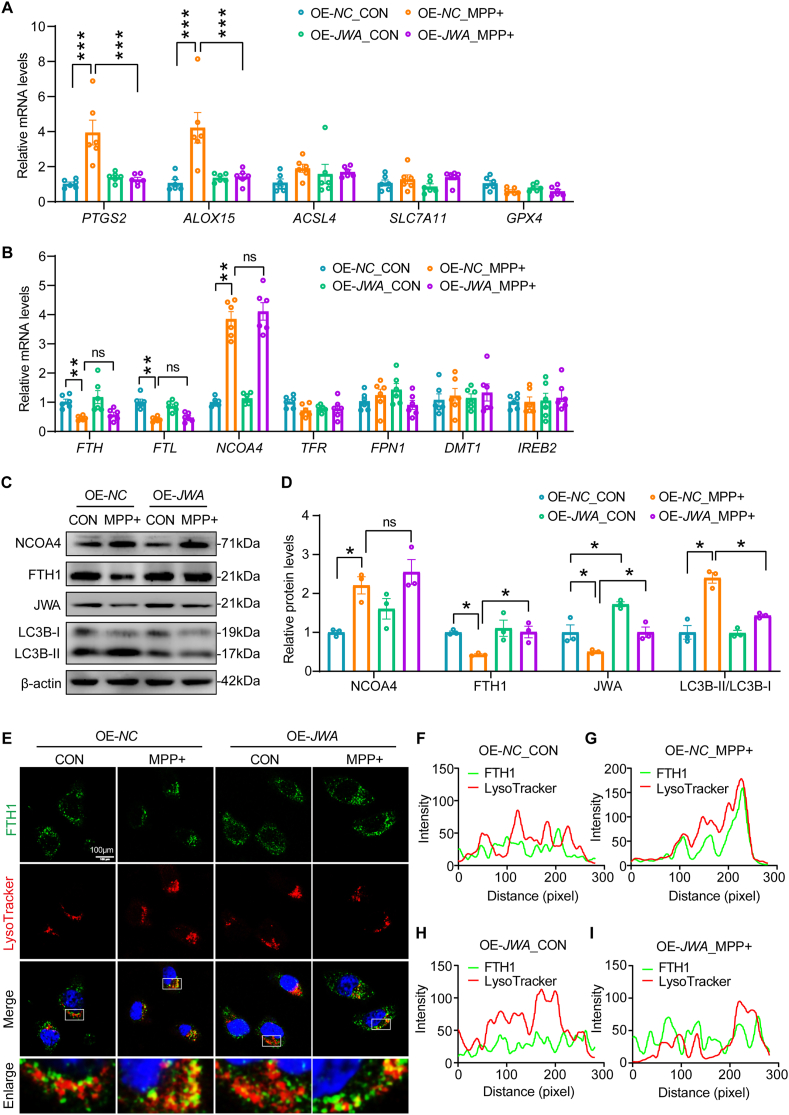
*JWA* overexpression hinders MPP + -induced ferritinophagy in SH-SY5Y cells. **(A**–**B)** RT-qPCR analysis of ferroptosis-related genes in WT and *JWA*-overexpressing SH-SY5Y cells exposed to MPP + or untreated. **p < 0.01, ***p < 0.001 by two-way ANOVA with Tukey's multiple comparisons test, n = 6, ns: no significance. **(C**–**D)** Western blotting analysis and quantification of ferritinophagy-related proteins in WT and *JWA*-overexpressing SH-SY5Y cells exposed to MPP + or untreated. *p < 0.05 by two-way ANOVA with Tukey's multiple comparisons test, n = 3, ns: no significance. **(E**–**I)** Immunofluorescence staining and quantification of colocalization of FTH1 and lysosomes in WT and *JWA*-overexpressing SH-SY5Y cells exposed to MPP + or untreated. Scale bar: 100 μm.

To unravel the mechanistic basis of JWA in ferroptosis regulation, we scrutinized the expression of ferroptosis-related genes in MPP + -treated SH-SY5Y cells. RT-qPCR analysis revealed a significant increase in the mRNA levels of ferroptosis indicators, namely *PTGS2* and *ALOX15*, upon MPP + treatment. Intriguingly, the overexpression of *JWA* markedly attenuated the expression of these two genes (Fig. 3A). Notably, *ACSL4*, *SCL7A11*, and *GPX4* did not exhibit any involvement in the inhibitory effect of ferroptosis by *JWA* overexpression (Fig. 3A). Subsequently, we assessed the expression of iron metabolism-associated genes. In the MPP + model, a significant reduction in the mRNA levels of *FTH* and *FTL*, accompanied by an upregulation in *NCOA4* mRNA levels, was observed. No alterations were noted in the mRNA levels of *TFR*, *FPN1*, *DMT1*, and *IREB2* (Fig. 3B). These findings suggest the potential involvement of iron metabolism, particularly ferritinophagy, in MPP + -induced ferroptosis of SH-SY5Y cells. Further investigation into proteins associated with ferritinophagy revealed that MPP + treatment induced ferritinophagy, evident by the upregulation of NCOA4 and downregulation of FTH1, coupled with LC3 turnover (Fig. 3C–D). While overexpression of *JWA* showed no impact on NCOA4 expression, it restored FTH1 expression and inhibited LC3 turnover, suggesting the inhibition of ferritinophagy (Fig. 3C–D). Immunostaining of FTH1 and lysotracker demonstrated that MPP + treatment induced the translocation of FTH1 to lysosomes, facilitating ferritinophagy. Conversely, overexpression of *JWA* prevented ferritinophagy by impeding the lysosomal translocation of FTH1 (Fig. 3E–I). Furthermore, our data showed increased expression of both IRP1 and IRP2 under MPP + treatment, in line with prior research [28], while no influence was observed upon *JWA* overexpression on IRP1 and IRP2 expression (Figs. S3A–D). These results suggest that the observed FTH1 upregulation under JWA overexpression largely stems from mitigating NCOA4-mediated FTH1 degradation rather than suppressing the IRP/IRE system. In contrast, we confirmed the impact of JWA on ferritinophagy in SH-SY5Y cells with *JWA* knockdown. The knockdown of *JWA* amplified ferritinophagy, exacerbating MPP + -induced ferroptosis in SH-SY5Y cells (Figs. S4A–D). This exacerbation was associated with the heightened promotion of lysosomal degradation of FTH1 (Figs. S4E–I). In conclusion, our findings indicate that JWA averts ferroptosis by inhibiting ferritinophagy in MPP + -treated SH-SY5Y cells.

### 3.4 JWA prevents ferritinophagy by binding to NCOA4 in MPP + -treated SH-SY5Y cells

**Fig. 4:**
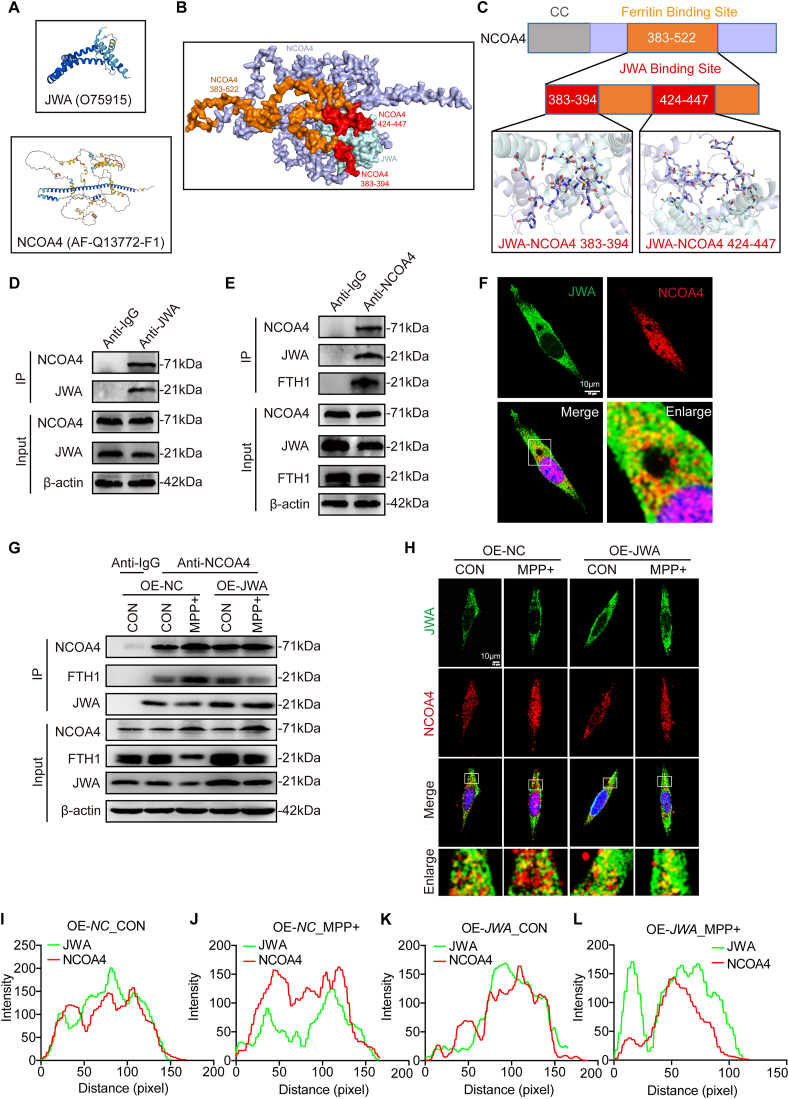
Overexpression of *JWA* interacts with NCOA4 to inhibits ferritinophagy in MPP + -treated SH-SY5Y cells. **(A)** The structures of JWA and NCOA4 obtained from the AlphaFold Protein Structure Database. **(B)** Simulation of the interaction between JWA and NCOA4 by the ZDOCK module of Discovery Studio 2019. **(C)** Illustration of the ferritin binding site and JWA binding site within NCOA4 by Pymol. CC: coiled coil domain. **(D**–**E)** Validation of JWA and NCOA4 interaction in the anti-JWA and anti-NCOA4 immunoprecipitants separately by Co-IP experiments in SH-SY5Y cells. **(F)** Validation of JWA and NCOA4 co-localization by immunofluorescence staining of JWA and NCOA4 in SH-SY5Y cells. Scale bar: 10 μm. **(G)** The competitive binding mode of JWA and FTH1 to NCOA4 observed by Co-IP experiments in WT and *JWA*-overexpressing SH-SY5Y cells exposed to MPP + or untreated. **(H**–**L)** Immunofluorescence staining and quantification of colocalization of JWA and NCOA4 in WT and *JWA*-overexpressing SH-SY5Y cells exposed to MPP + or untreated. Scale bar: 10 μm. IP: immunoprecipitation.

Given that NCOA4 has been recognized as a selective cargo receptor for the autophagic turnover of ferritin [29], we next explored the interaction between JWA and NCOA4 in SH-SY5Y cells. The protein structures of JWA and NCOA4 were obtained from the AlphaFold Protein Structure Database (https://alphafold.ebi.ac.uk/), and the ZDOCK module of Discovery Studio 2019 was employed to simulate the interaction (Fig. 4A–B). Remarkably, the FTH1 binding domain (Ferritin binding site), reported to be within the 383–522 amino acids of NCOA4 [30], contained two JWA binding sites: 383-394aa and 424-447aa of NCOA4 (Fig. 4C). This suggests that the inhibition of ferritinophagy by JWA may be attributed to its occupation of the FTH1 binding site on NCOA4. Subsequently, we validated the interaction between JWA and NCOA4 through Co-IP experiments. NCOA4 was detected in the Anti-JWA immunoprecipitants, while both JWA and FTH1 were detected in the Anti-NCOA4 immunoprecipitants (Fig. 4D–E). Immunofluorescence staining of JWA and NCOA4 revealed colocalization in SH-SY5Y cells (Fig. 4F). Moreover, in the MPP + model, an augmented interaction between NCOA4 and FTH1 was observed, indicating an acceleration of ferritinophagy. Conversely, overexpression of *JWA* increased the binding of JWA to NCOA4, thereby reducing ferritinophagy by impeding the binding of FTH1 to NCOA4 (Fig. 4G). The enhanced co-localization of JWA to NCOA4 was further visualized in JWA-overexpressing SH-SY5Y cells through immunofluorescence staining of JWA and NCOA4 in the MPP + model (Fig. 4H-L). Thus, our findings unveil that JWA hinders ferritinophagy by binding to NCOA4, preventing NCOA4-mediated FTH1 degradation.

### 3.5 JAC4 treatment enhances the resistance of neurons to MPP + -induced ferroptosis

**Fig. 5:**
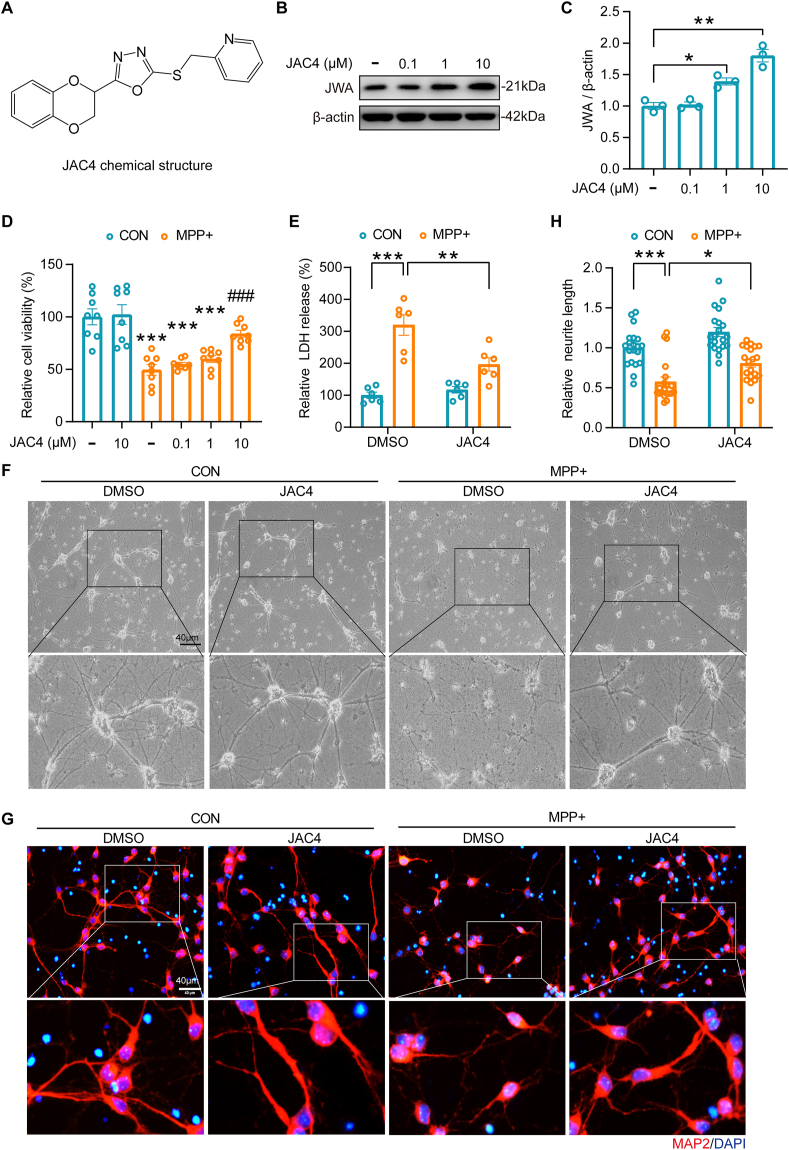
JAC4 treatment attenuates MPP + -induced injury in primary neurons by upregulating JWA expression. **(A)** The chemical structure of JAC4. **(B–C)** Western blotting analysis and quantification of JWA expression by JAC4 treatment for 24 h in primary neurons. *p < 0.05, **p < 0.01 by one-way ANOVA with Tukey's multiple comparisons test, n = 3. **(D)** Assessment of cell viability in primary neurons pre-treated with JAC4 for 1 h, followed by 24-h MPP + exposure or untreated. ***p < 0.001 vs. CON group, ###p < 0.001 vs. MPP + group by one-way ANOVA with Tukey's multiple comparisons test, n = 8. **(E)** Measurement of LDH release in primary neurons pre-treated with JAC4 (10 μM) for 1 h, followed by 24-h MPP + exposure or untreated. **p < 0.01, ***p < 0.001 by two-way ANOVA with Tukey's multiple comparisons test, n = 6. **(F)** Visualization of primary neuron morphology following JAC4 pretreatment (10 μM, 1 h) and subsequent 24-h MPP + exposure or untreated. Scale bar: 40 μm. **(G**–**H)** Immunofluorescence staining of MAP2 and quantification of neurite length in primary neurons pre-treated with JAC4 (10 μM) for 1 h, followed by 24-h MPP + exposure or untreated. *p < 0.05, ***p < 0.001 by two-way ANOVA with Tukey's multiple comparisons test, n = 20, scale bar: 40 μm.

JAC4, a compound synthesized by our collaborator Prof. Zhou in his laboratory to increase JWA expression [21], demonstrated a dose-dependent upregulation of JWA expression in primary neurons (Fig. 5A–C). Importantly, treatment with JAC4 at 10 μM exhibited protective effects against MPP + -induced neuronal damage, as evidenced by an increase in cell viability and a decrease in LDH release (Fig. 5D–E). Morphologically, JAC4 treatment ameliorated the loss of neurites in MPP + -treated primary neurons (Fig. 5F–H), highlighting the neuroprotective potential of JAC4 in MPP + -induced neuronal injury.

**Fig. 6:**
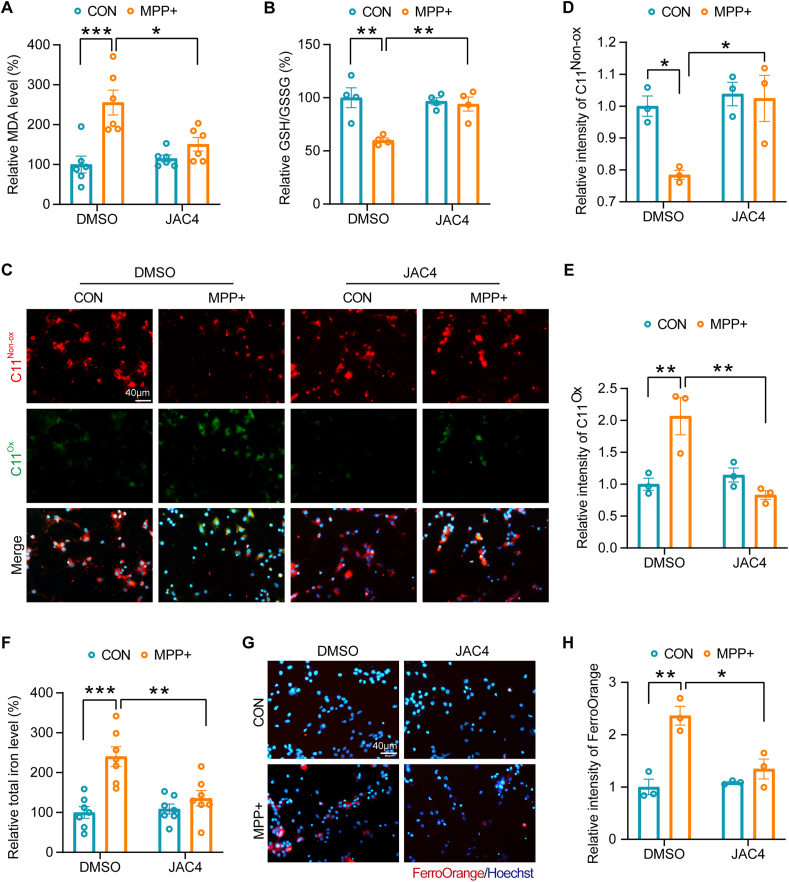
JAC4 treatment enhances the resistance of MPP + -treated primary neurons to ferroptosis. **(A)** Measurement of MDA levels in primary neurons pre-treated with JAC4 (10 μM) for 1 h, followed by 24-h MPP + exposure or untreated. *p < 0.05, ***p < 0.001 by two-way ANOVA with Tukey's multiple comparisons test, n = 6. **(B)** Assessment of the GSH/GSSG ratio in primary neurons pre-treated with JAC4 (10 μM) for 1 h, followed by 24-h MPP + exposure or untreated. **p < 0.01 by two-way ANOVA with Tukey's multiple comparisons test, n = 4. **(C**–**E)** Staining and quantification of the reduced and oxidized forms of C11-BODIPY in primary neurons pre-treated with JAC4 (10 μM) for 1 h, followed by 24-h MPP + exposure or untreated. *p < 0.05, **p < 0.01 by two-way ANOVA with Tukey's multiple comparisons test, n = 3, scale bar: 40 μm. **(F)** Measurement of total iron levels in primary neurons pre-treated with JAC4 (10 μM) for 1 h, followed by 24-h MPP + exposure or untreated. **p < 0.01, ***p < 0.001 by two-way ANOVA with Tukey's multiple comparisons test, n = 7. **(G**–**H)** FerroOrange staining and quantification of ferrous ion in primary neurons pre-treated with JAC4 (10 μM) for 1 h, followed by 24-h MPP + exposure or untreated. *p < 0.05, **p < 0.01 by two-way ANOVA with Tukey's multiple comparisons test, n = 3, scale bar: 40 μm.

Next, we investigated the impact of JAC4 treatment on ferroptosis in primary neurons within the MPP + model. Remarkably, JAC4 treatment restored the increased MDA level and reduced ratio of GSH/GSSG in MPP + -treated primary neurons (Fig. 6A–B). Subsequently, using the C11-BODIPY probe, we assessed lipid peroxidation in primary neurons. MPP + treatment induced substantial oxidation of C11-BODIPY accompanied by a decrease in the reduced form. Conversely, JAC4 treatment increased the reduced form and decreased the oxidized form of C11-BODIPY, indicative of the inhibition of lipid peroxidation in the MPP + model (Fig. 6C–E). Moreover, JAC4 treatment also reduced the total iron level and Fe^2+^ ions in primary neurons subjected to MPP+, as visualized by FerroOrange staining (Fig. 6F–H). Then, we evaluated the impact of JAC4 on iron homeostasis and lipid peroxidation in differentiated SH-SY5Y cells, which are widely utilized in recapitulating the characteristics of DA neurons [[31], [32], [33]]. Our data showed that JAC4 treatment reduces lipid peroxidation and iron accumulation in MPP + -stimulated differentiated SH-SY5Y cells (Figs. S5A–E). Collectively, our findings demonstrate that JAC4 treatment effectively restrains neuronal ferroptosis through the upregulation of JWA expression.

### 3.6 JAC4 treatment mitigates PD pathology through inhibition of ferroptosis in the MPTP-induced PD mice

**Fig. 7:**
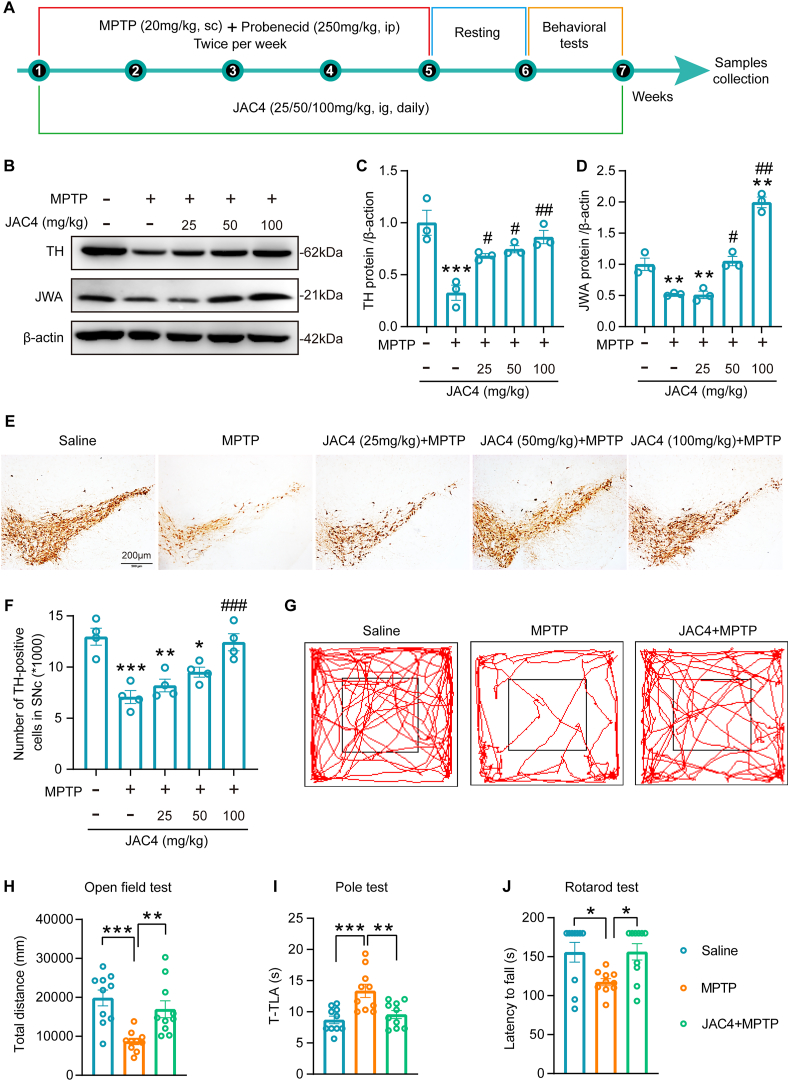
Treatment with JAC4 in a dose-dependent manner improves PD pathology in MPTP mice **(A)** Schematic representation of the experimental design. **(B**–**D)** Western blotting analysis and quantification of the expression levels of TH and JWA in the midbrain of mice treated with JAC4 in the MPTP model. **p < 0.01, ***p < 0.001 vs. Saline group, #p < 0.05, ##p < 0.01 vs. MPTP group by one-way ANOVA with Tukey's multiple comparisons test, n = 3. **(E**–**F)** Immunohistochemical staining and quantification of TH-positive cells in the SNc region of mice treated with JAC4 in the MPTP model. *p < 0.05, **p < 0.01, ***p < 0.001 vs. Saline group, ###p < 0.001 vs. MPTP group by one-way ANOVA with Tukey's multiple comparisons test, n = 4, scale bar: 200 μm. **(G**–**H)** The representative travel path and quantification of total distance in the open field test. **p < 0.01, ***p < 0.001 by one-way ANOVA with Tukey's multiple comparisons test, n = 10. JAC4 treatment: 100 mg/kg. **(I)** Recording of the time spent climbing off the pole in the pole test. **p < 0.01, ***p < 0.001 by one-way ANOVA with Tukey's multiple comparisons test, n = 10. JAC4 treatment: 100 mg/kg. **(J)** Recording of the latency to fall in the rotarod test. *p < 0.05 by one-way ANOVA with Tukey's multiple comparisons test, n = 10. JAC4 treatment: 100 mg/kg.

To assess the therapeutic potential of JAC4 treatment in the progression of PD, we utilized the MPTP-induced mouse model of PD (Fig. 7A). Our results revealed that JAC4 treatment dose-dependently increased the expression of TH and JWA in the midbrain of PD mice (Fig. 7B–D). Importantly, treatment with JAC4 at 100 mg/kg significantly rescued the loss of DA neurons in the SNc region of PD mice (Fig. 7E–F). In behavioral tests, JAC4 treatment increased the travel distance of PD mice in the open field test (Fig. 7G–H), reduced the time spent climbing off the pole in the pole test (Fig. 7I), and restored the decreased latency to fall in the rotarod test (Fig. 7J). Subsequently, neurotransmitter levels and their metabolites in the striatum were measured by HPLC. The levels of dopamine and its metabolites, including DOPAC, 3-MT, and HVA, were significantly increased in PD mice following JAC4 treatment (Figs. S6A–D). However, there were no alterations in the levels of 5-HT and 5-HIAA in this context (Figs. S6E–F). These results suggest that JAC4 treatment has therapeutic potential in mitigating PD pathology and improving movement symptoms.

**Fig. 8:**
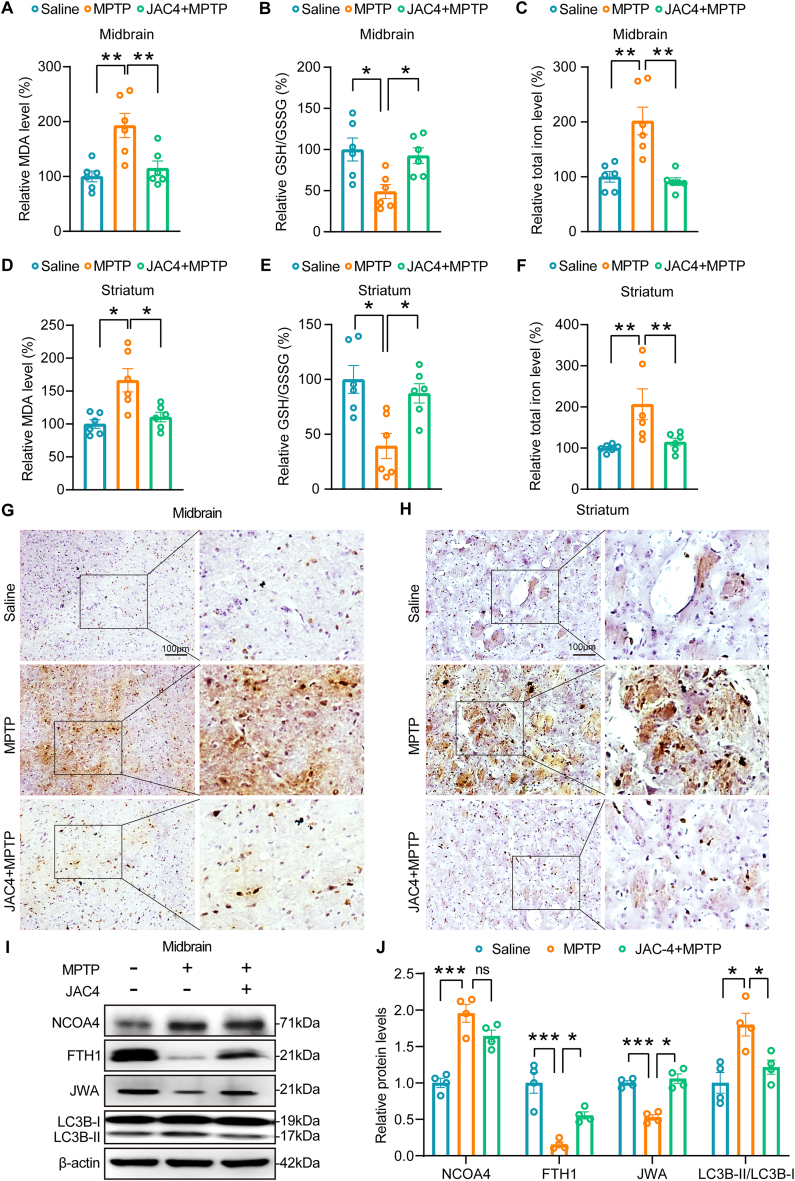
JAC4 administration strengthens the resilience of MPTP mice against ferroptosis. **(A, D)** Measurement of MDA levels in the midbrain and striatum of mice treated with JAC4 (100 mg/kg) in the MPTP model. *p < 0.05, **p < 0.01 by one-way ANOVA with Tukey's multiple comparisons test, n = 6. **(B, E)** Evaluation of the GSH/GSSG ratio in the midbrain and striatum of mice treated with JAC4 (100 mg/kg) in the MPTP model. *p < 0.05 by one-way ANOVA with Tukey's multiple comparisons test, n = 6. **(C, F)** Quantification of iron levels in the midbrain and striatum of mice treated with JAC4 (100 mg/kg) in the MPTP model. **p < 0.01 by one-way ANOVA with Tukey's multiple comparisons test, n = 6. **(G**–**H)** Perls Prussian blue staining of brain slices in the midbrain and striatum of mice treated with JAC4 (100 mg/kg) in the MPTP model. Scale bar: 100 μm. **(I**–**J)** Western blotting analysis and quantification of ferritinophagy-related proteins in the midbrain of mice treated with JAC4 (100 mg/kg) in the MPTP model. *p < 0.05, ***p < 0.001 by one-way ANOVA with Tukey's multiple comparisons test, n = 4, ns: no significance. (For interpretation of the references to color in this figure legend, the reader is referred to the Web version of this article.)

In the midbrain of MPTP-induced PD mice, there was an increase in MDA production and a decrease in the ratio of GSH to GSSG, indicative of oxidative stress and lipid peroxidation. JAC4 administration effectively restored these changes (Fig. 8A–B). Notably, iron accumulation in the midbrain of PD mice was also reduced by JAC4 treatment (Fig. 8C). Consistent with these findings, similar trends were observed in the striatum of PD mice, where JAC4 treatment mitigated MDA increase, restored GSH/GSSG ratio, and reduced iron accumulation (Fig. 8D–F). Perls Prussian blue staining further confirmed a significant reduction in iron accumulation by JAC4 administration in MPTP-induced PD mice (Fig. 8G–H). Western blotting analysis demonstrated that JAC4 administration remarkably impeded ferroptosis by blunting the ferritinophagy pathway in the midbrain and striatum of PD mice (Fig. 8I–J and Figs. S7A–B). In conclusion, the therapeutic potential of JAC4 treatment for PD is attributed to the upregulation of JWA, which in turn dampens ferritinophagy-induced ferroptosis.

## 4 Discussion

Parkinson's disease (PD) is characterized by the progressive loss of DA neurons in the SNc region, leading to compromised dopamine supply in the nigrostriatal pathway and subsequent motor dysfunction. Extensive research has focused on the intricate mechanisms governing DA neuronal death, with ferroptosis emerging as a key player. Our study contributes a novel perspective to the current understanding by introducing JWA as a previously unrecognized regulatory entity effectively mitigating ferroptosis in DA neurons. Notably, JWA achieves this protective effect by impeding NCOA4-mediated ferritinophagy, unveiling a critical regulatory mechanism in the context of PD. Furthermore, the synthesis of JAC4, a small molecular drug designed to elevate JWA expression, presents itself as a therapeutic intervention with the potential to curtail ferritinophagy, thereby demonstrating promise in the treatment of PD. In essence, our work not only identifies JWA as a new negative regulator of ferroptosis in the degeneration of DA neurons but also illuminates a prospective target within the ferritinophagy pathway for innovative PD therapies.

The association between iron dysregulation and lipid peroxidation in PD patients and animal models predates the formal recognition of ferroptosis, as evidenced by earlier studies [[6], [7], [8]]. With the subsequent definition of ferroptosis [5], investigations have increasingly linked the degeneration of DA neurons to this intricate process in the progression of PD [9,10]. A notable intervention in iron dynamics, the iron chelator deferiprone, originally approved for treating transfusion-dependent thalassemia, has demonstrated a paradoxical impact on PD. While effectively reducing iron content, its exacerbation of parkinsonism in a phase 2 randomized clinical trial underscores the intricate and multifaceted nature of iron dynamics in PD [12]. Our study builds upon these insights, observing iron accumulation and lipid peroxidation in DA neurons within the PD context. Crucially, both *JWA* overexpression and JAC4 administration emerged as promising strategies to restore iron and MDA levels, effectively mitigating ferroptosis in DA neurons. These findings suggest that achieving a delicate balance in iron content and lipid peroxidation products, rather than simply removing them, may hold the key to therapeutic advancements in PD.

*JWA*, initially identified as an all-trans retinoic acid-responsive gene, has garnered attention for its multifaceted role in cellular homeostasis [13]. Previous studies have highlighted its involvement in diminishing ROS production and augmenting glutathione and glutathione peroxidase levels, implicating a potential regulatory role in ferroptosis [14,15]. Furthermore, our group has previously underscored the indispensability of JWA for the survival of DA neurons in the context of PD [16,17]. Building upon this foundational understanding, our present investigation unveils synchronized expression patterns of JWA and TH in PD patients and mice, emphasizing the pivotal role of JWA in maintaining DA neuronal survival. To delineate the regulatory mechanisms of ferroptosis by JWA, we employed SH-SY5Y cell lines with manipulated *JWA* expression. Our findings suggest that JWA functions as a negative regulator of ferroptosis, specifically by restoring iron content and MDA levels. While the intricate processes governing ferroptosis encompass redox homeostasis, iron metabolism, and lipid metabolism [34], our study pinpointed the pivotal role of JWA in iron dynamics. Notably, the prevention of ferroptosis by JWA was primarily attributed to the equilibrium in iron metabolism. The overexpression of *JWA* successfully restored the labile iron pool (LIP) by impeding ferritinophagy-induced iron accumulation. Conversely, *JWA* knockdown amplified ferritinophagy, thereby inciting the Fenton reaction and facilitating ferroptosis. Interestingly, we found that both the mRNA levels of *FTH*/*FTL* and the protein level of FTH1 were reduced in the MPP + model, which may be attributed the decreased Nrf2 expression as reported previously [35,36]. However, *JWA* overexpression served to increase the protein level of FTH1, with no corresponding changes to the mRNA levels of *FTH*/*FTL*. The IRP/IRE system maintains cellular iron homeostasis by curbing ferritin expression at the transcriptional level [37]. Our data showed increased expression of both IRP1 and IRP2 under MPP + treatment, in line with prior research [28], while no influence was observed upon *JWA* overexpression on IRP1 and IRP2 expression. To unravel the intricate regulation of JWA in ferritinophagy, we delved into the interaction between JWA and NCOA4, a selective cargo receptor orchestrating the autophagic turnover of ferritin [29]. Our results provide compelling evidence that JWA exerts its regulatory process by occupying the FTH1 binding site of NCOA4, effectively obstructing NCOA4-mediated ferritinophagy. This intricate interplay positions JWA as a novel negative regulator of ferroptosis through the targeted inhibition of NCOA4-mediated ferritinophagy.

To evaluate the potential of JWA as a therapeutic target for PD, we employed JAC4, a compound designed to activate JWA by enhancing its expression [21]. JAC4 treatment demonstrated effectiveness in elevating JWA expression across various tissues, encompassing the intestine, lung, and brain [21,38,39]. In line with these observations, our study revealed a significant increase in JWA expression in primary neurons and the midbrain of mice upon JAC4 treatment. By augmenting JWA expression, JAC4 treatment exhibited a notable capacity to fortify the resistance of primary neurons exposed to MPP + against ferroptosis. Moreover, the administration of JAC4 yielded promising outcomes in vivo, as it not only reduced the loss of DA neurons in the SNc region but also ameliorated motor symptoms in PD mice. The neuroprotective mechanism attributed to JAC4 treatment was associated with the restoration of iron content and the mitigation of lipid peroxidation, achieved through the inhibition of ferritinophagy. These findings collectively position JWA as a promising therapeutic target for PD, offering a potential avenue to impede DA neuronal ferroptosis.

**Fig. 9:**
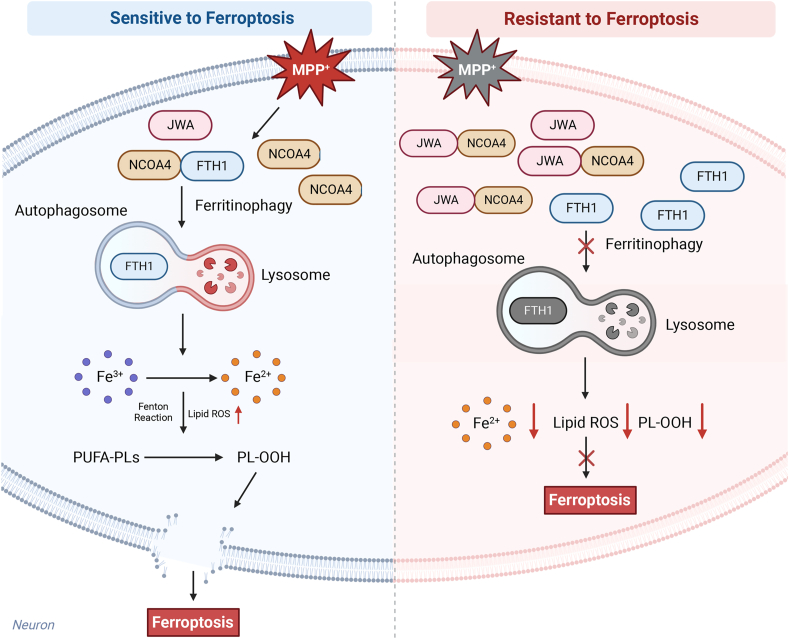
The schematic illustration delineates the proposed mechanism through which JWA operates to suppress ferroptosis in PD. In the PD context, reduced JWA expression facilitates the interaction between FTH1 and NCOA4, intensifying ferritinophagy. Elevated levels of ferrous ions catalyze the Fenton reaction and instigate lipid peroxidation, ultimately initiating ferroptosis in DA neurons. The heightened expression of JWA, whether induced through genetic manipulation or pharmacological intervention, disrupts NCOA4-mediated ferritinophagy by occupying the FTH1 binding site within NCOA4. Consequently, JWA functions as a negative regulator of ferroptosis, reinstating the labile iron pool (LIP) and thereby safeguarding DA neurons during the progression of PD.

In summary, our study sheds light on the susceptibility of DA neurons to ferroptosis in the progression of PD, attributed to the heightened NCOA4-mediated ferritinophagy. The pivotal role of ferritinophagy in orchestrating ferroptotic cascades unveils a previously unexplored facet of DA neuronal vulnerability in PD. Leveraging this insight, our investigation introduces JWA as a novel negative regulator, bolstering the resistance of DA neurons to ferroptosis. By binding to NCOA4 and impeding NCOA4-mediated ferritinophagy, JWA emerges as a crucial guardian against the degradation of FTH1, thereby mitigating the ferroptotic demise of DA neurons (Fig. 9). The translational implications of our findings are profound, suggesting that strategies focused on increasing JWA expression, exemplified by JAC4 treatment, hold therapeutic promise for PD. The envisioned therapeutic approach involves fortifying the intrinsic defense mechanisms against ferroptosis, thus potentially offering a transformative strategy in PD management. However, the detailed molecular underpinnings of JWA's regulation of ferritinophagy remain a ripe area for exploration, inviting future investigations to unravel the intricate network of molecular players, such as the potential interplay of the autophagy-lysosomal pathway with ferritinophagy. In addition, the translational potential of JAC4 demands rigorous scrutiny, necessitating comprehensive assessments of safety and efficacy in preclinical and clinical settings. These endeavors will pave the way for the potential translation of JAC4 into a viable therapeutic avenue for PD. Although challenges lie ahead, our work not only advances the comprehension of DA neuronal ferroptosis but also signifies a significant stride in therapeutic strategy development. The exploration of ferritinophagy as a target opens new avenues for further research, promising a deeper understanding and innovative interventions in the landscape of neurodegenerative disorders.

## Data and materials availability

All data essential for evaluating the conclusions presented in the paper are provided in the paper and/or the Supplementary Materials. Additional data can be made available by contacting the authors directly.

## CRediT authorship contribution statement

**Xinxin Zhao:** Visualization, Methodology, Investigation, Formal analysis, Data curation. **Zhengwei Kang:** Resources, Methodology, Data curation. **Ruixue Han:** Methodology, Investigation, Data curation. **Min Wang:** Validation, Methodology. **Yueping Wang:** Validation, Methodology. **Xin Sun:** Resources, Investigation. **Cong Wang:** Software, Resources. **Jianwei Zhou:** Resources, Conceptualization. **Lei Cao:** Writing – original draft, Visualization, Supervision, Funding acquisition, Conceptualization. **Ming Lu:** Writing – review & editing, Supervision, Funding acquisition, Conceptualization.

## Declaration of competing interest

The authors declare no competing interests.

## Data Availability

Data will be made available on request.
