# Supplementary material for: JWA binding to NCOA4 alleviates degeneration in dopaminergic neurons through suppression of ferritinophagy in Parkinson's disease
Source: Redox Biol. 2024 May 13;73:103190. doi: 10.1016/j.redox.2024.103190 (PMC11109895; doi:10.1016/j.redox.2024.103190)
Supplement: Multimedia component 1 [file mmc1.docx]

**JWA binding to NCOA4 alleviates degeneration in dopaminergic neurons through suppression of ferritinophagy in Parkinson’s disease**

Xinxin Zhao ^a,1^, Zhengwei Kang ^a,1^, Ruixue Han ^a,1^, Min Wang ^a^, Yueping Wang ^a^, Xin Sun ^a^, Cong Wang ^a^, Jianwei Zhou ^b,c^, Lei Cao ^a,c, *^, Ming Lu ^a,c,*^

^a^ Jiangsu Key Laboratory of Neurodegeneration, Department of Pharmacology, Nanjing Medical University, Nanjing 211166, China;

^b^ Department of Molecular Cell Biology & Toxicology, Center for Global Health, School of Public Health, Nanjing Medical University, Nanjing 211166, China;

^c^ Changzhou Second People's Hospital, Changzhou Medical Center, Nanjing Medical University, Changzhou 213000, China

^*^ Corresponding author. Jiangsu Key Laboratory of Neurodegeneration, Department of Pharmacology, Nanjing Medical University, Nanjing 211166, China; Changzhou Second People's Hospital, Changzhou Medical Center, Nanjing Medical University, Changzhou 213000, China.

*E*-mail addresses: lum@njmu.edu.cn (Ming Lu), leicao@njmu.edu.cn (Lei Cao)

^1^ These authors contributed equally to this work.

**Supplementary figures and figure legends**


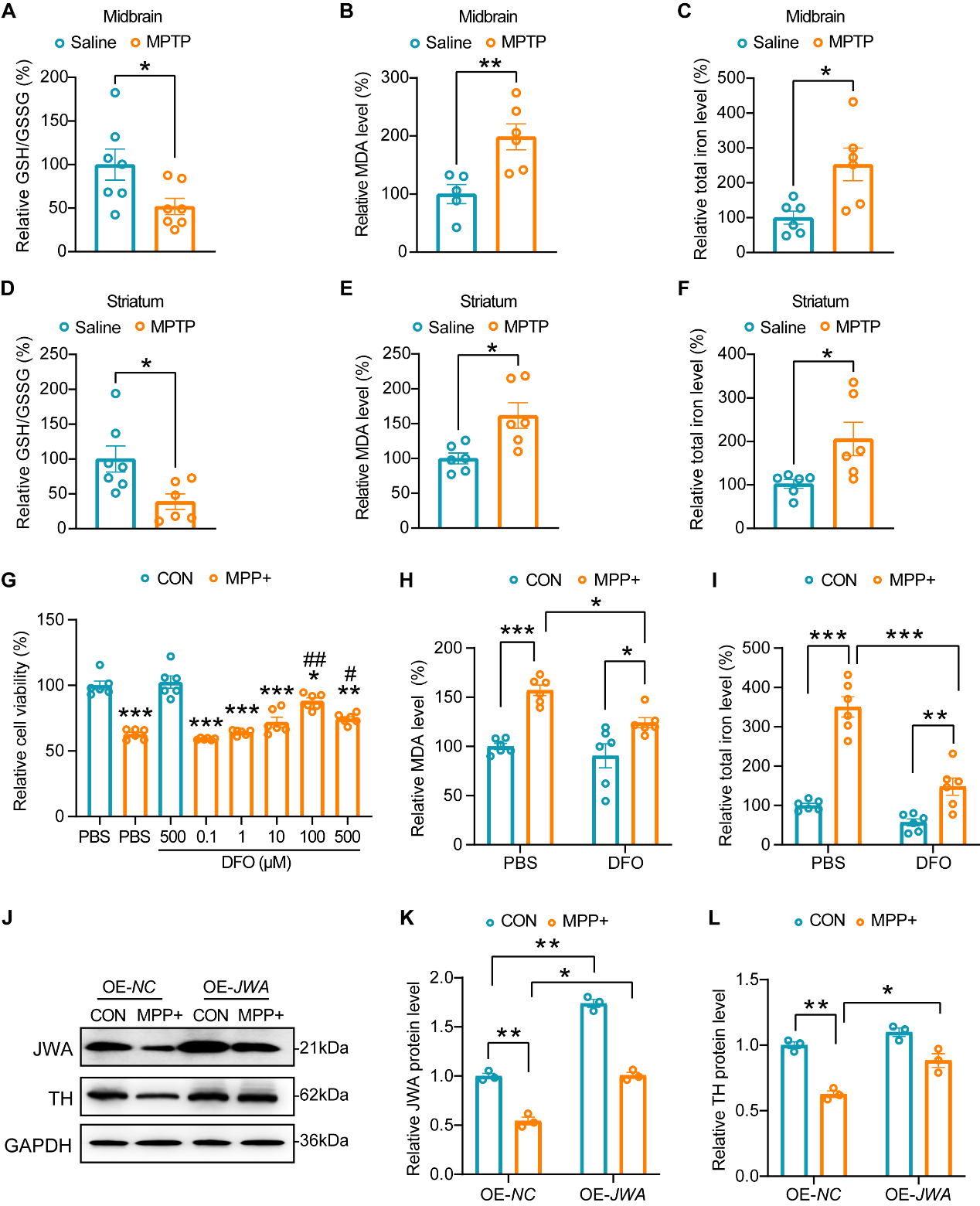


**Figure S1. Ferroptosis is induced in the MPTP/MPP+-based model of PD.**

**(A, D)** Measurement of the GSH/GSSG ratio in the midbrain and striatum of mice subjected to Saline and MPTP. *p<0.05 by unpaired *t*-test, n=6-7. **(B, E)** Determination of MDA levels in the midbrain and striatum of mice subjected to Saline and MPTP. *p<0.05, **p<0.01 by unpaired *t*-test, n=5-6. **(C, F)** Evaluation of iron levels in the midbrain and striatum of mice subjected to Saline and MPTP. *p<0.05 by unpaired *t*-test, n=6. **(G)** Assessment of cell viability in SH-SY5Y cells pre-treated with deferiprone (DFO, 0.1-500 μM) for 1 hour, followed by 24-hour MPP+ treatment or untreated. *p<0.05, **p<0.01, ***p<0.001 vs. CON group, #p<0.05, ##p<0.01 vs. MPP+ group by one-way ANOVA with Tukey’s multiple comparisons test, n=6. **(H)** Determination of MDA levels in SH-SY5Y cells pre-treated with deferiprone (DFO, 100 μM) for 1 hour, followed by 24-hour MPP+ treatment or untreated. *p<0.05, ***p<0.001 by two-way ANOVA with Tukey’s multiple comparisons test, n=6. **(I)** Evaluation of iron levels in SH-SY5Y cells pre-treated with deferiprone (DFO, 100 μM) for 1 hour, followed by 24-hour MPP+ treatment or untreated. **p<0.01, ***p<0.001 by two-way ANOVA with Tukey’s multiple comparisons test, n=6. **(J-L)** Representative blots and quantitative analysis of JWA and TH in *JWA* overexpression SH-SY5Y cells treated with MPP+. *p<0.05, **p<0.01 by two-way ANOVA with Tukey’s multiple comparisons test, n=3.


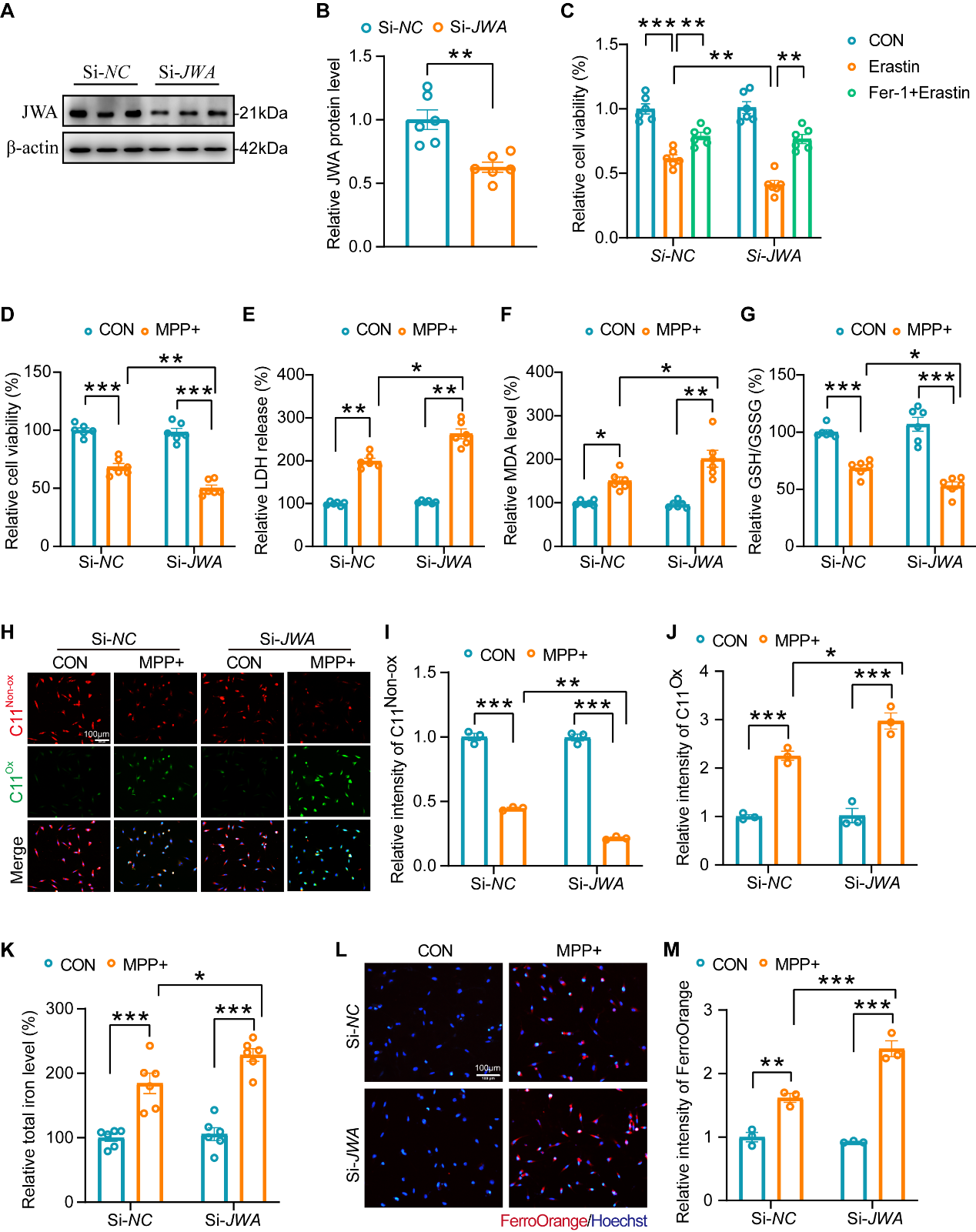


**Figure S2. Knockdown of *JWA* augments the susceptibility of MPP+-treated SH-SY5Y cells to ferroptosis.**

**(A-B)** Evaluation of *JWA* knockdown efficiency in SH-SY5Y cells through Western blotting analysis and quantification. **p<0.01 by unpaired *t*-test, n=6. **(C)** Effect of *JWA* knockdown on Erastin-induced ferroptosis model. **p<0.01, ***p<0.001 by two-way ANOVA with Tukey’s multiple comparisons test, n=6. **(D)** Assessment of cell viability via CCK-8 assay in WT and *JWA* knockdown SH-SY5Y cells exposed to MPP+ or untreated. **p<0.01, ***p<0.001 by two-way ANOVA with Tukey’s multiple comparisons test, n=6. **(E)** Measurement of LDH release in WT and *JWA* knockdown SH-SY5Y cells exposed to MPP+ or untreated. *p<0.05, **p<0.01 by two-way ANOVA with Tukey’s multiple comparisons test, n=6. **(F)** Quantification of MDA levels in WT and *JWA* knockdown SH-SY5Y cells exposed to MPP+ or untreated. *p<0.05, **p<0.01 by two-way ANOVA with Tukey’s multiple comparisons test, n=6. **(G)** Assessment of the GSH/GSSG ratio in WT and *JWA* knockdown SH-SY5Y cells exposed to MPP+ or untreated. *p<0.05, ***p<0.001 by two-way ANOVA with Tukey’s multiple comparisons test, n=6. **(H-J)** Staining and quantification of the reduced and oxidized forms of C11-BODIPY in WT and *JWA* knockdown SH-SY5Y cells exposed to MPP+ or untreated. *p<0.05, **p<0.01, ***p<0.001 by two-way ANOVA with Tukey’s multiple comparisons test, n=3, scale bar: 100 μm. **(K)** Measurement of total iron levels in WT and *JWA* knockdown SH-SY5Y cells exposed to MPP+ or untreated. *p<0.05, ***p<0.001 by two-way ANOVA with Tukey’s multiple comparisons test, n=6. **(L-M)** FerroOrange staining and quantification of ferrous ion in WT and *JWA* knockdown SH-SY5Y cells exposed to MPP+ or untreated. **p<0.01, ***p<0.001 by two-way ANOVA with Tukey’s multiple comparisons test, n=3, scale bar: 100 μm.


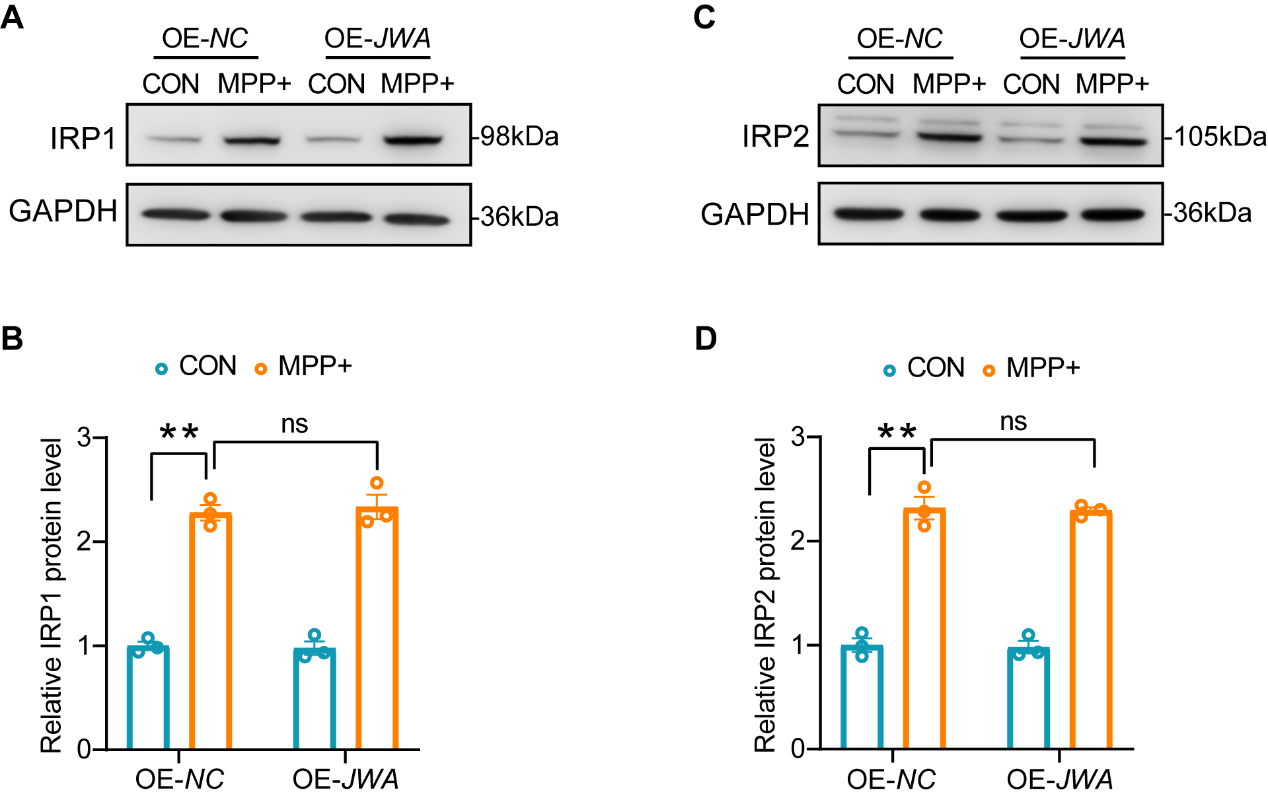


**Figure S3. Overexpression of *JWA* has no influence on MPP+-induced upregulation of IRP1 and IRP2 in SH-SY5Y cells.**

**(A-B)** Representative blots and quantification of IRP1 in SH-SY5Y cells. **p<0.01 by two-way ANOVA with Tukey’s multiple comparisons test, n=3, ns: no significance. **(C-D)** Representative blots and quantification of IRP2 in SH-SY5Y cells. **p<0.01 by two-way ANOVA with Tukey’s multiple comparisons test, n=3, ns: no significance.


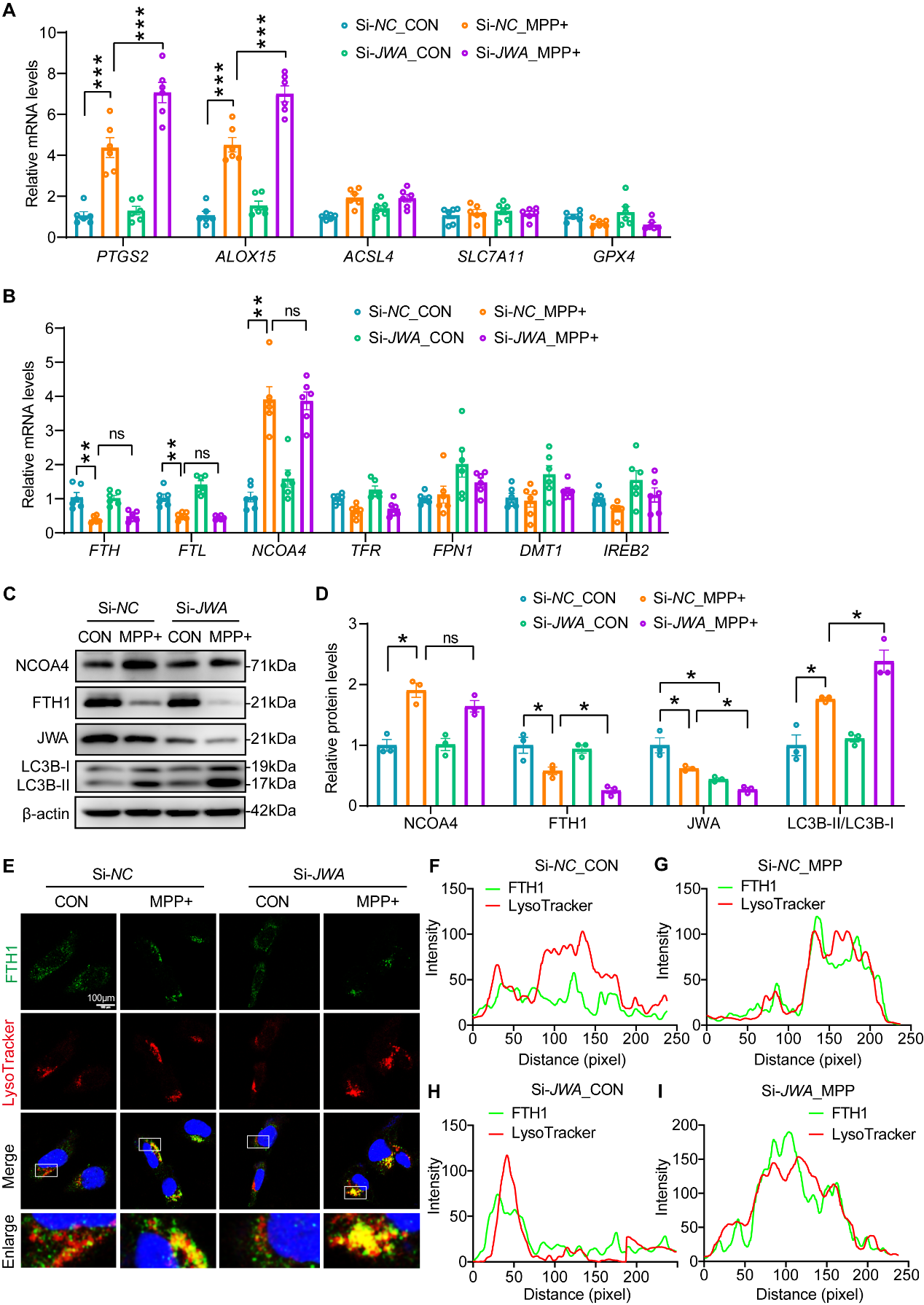


**Figure S4. Knockdown of *JWA* boosts MPP+-induced ferritinophagy in SH-SY5Y cells.**

**(A-B)** RT-qPCR analysis of ferroptosis-related genes in WT and *JWA* knockdown SH-SY5Y cells exposed to MPP+ or untreated. **p<0.01, ***p<0.001 by two-way ANOVA with Tukey’s multiple comparisons test, n=6, ns: no significance. **(C-D)** Western blotting analysis and quantification of ferritinophagy-related proteins in WT and *JWA* knockdown SH-SY5Y cells exposed to MPP+ or untreated. *p<0.05 by two-way ANOVA with Tukey’s multiple comparisons test, n=3, ns: no significance. **(E-I)** Immunofluorescence staining and quantification of colocalization of FTH1 and lysosomes in WT and *JWA* knockdown SH-SY5Y cells exposed to MPP+ or untreated. Scale bar: 100 μm.


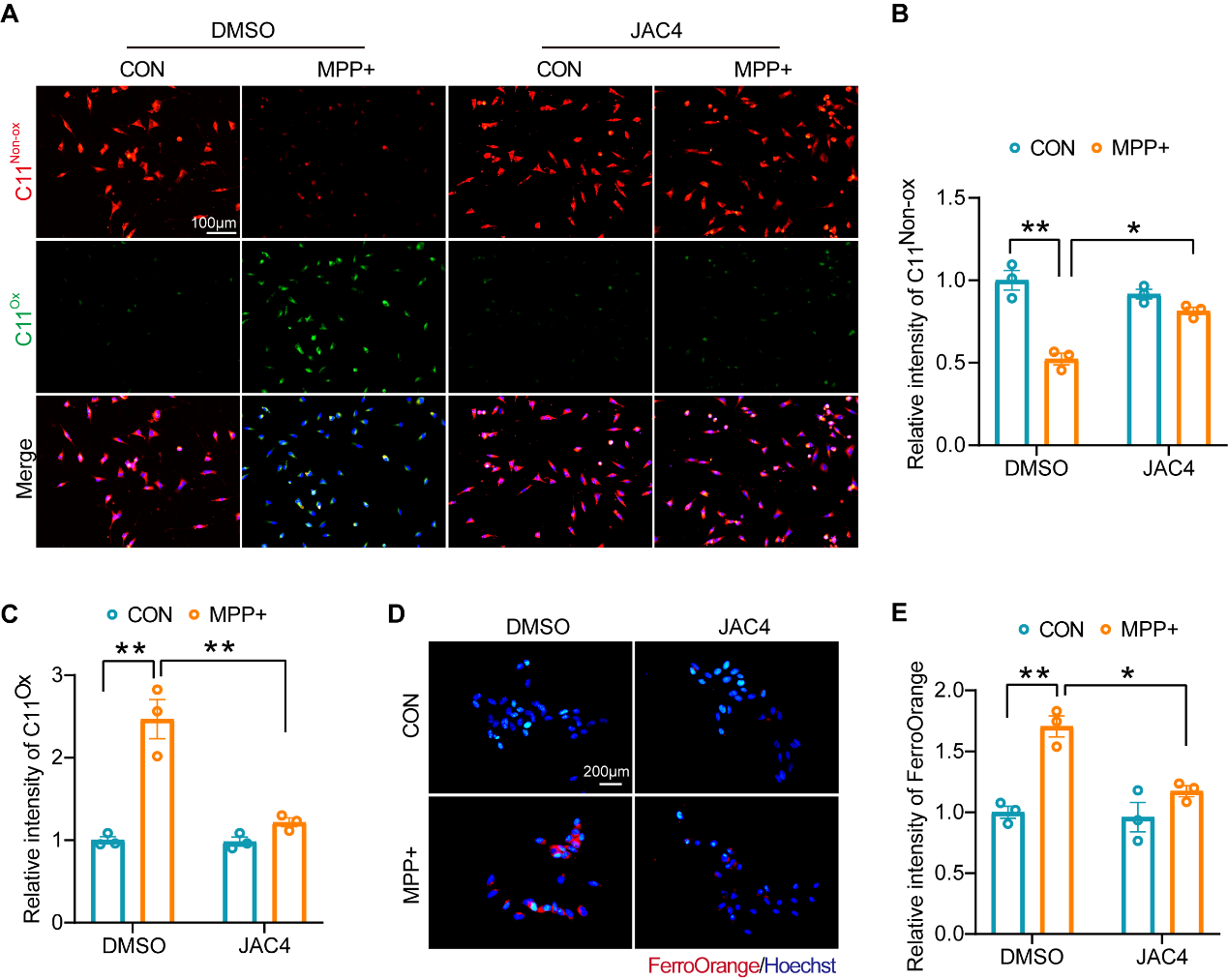


**Figure S5. JAC4 treatment inhibits lipid peroxidation and iron accumulation in MPP+-treated SH-SY5Y cells.**

**(A-C)** Staining and quantification of the reduced and oxidized forms of C11-BODIPY in JAC4-treated SH-SY5Y cells exposed to MPP+ or untreated. *p<0.05, **p<0.01 by two-way ANOVA with Tukey’s multiple comparisons test, n=3, scale bar: 100 μm. **(D-E)** FerroOrange staining and quantification of ferrous ion in JAC4-treated SH-SY5Y cells exposed to MPP+ or untreated. *p<0.05, **p<0.01 by two-way ANOVA with Tukey’s multiple comparisons test, n=3, scale bar: 200 μm.


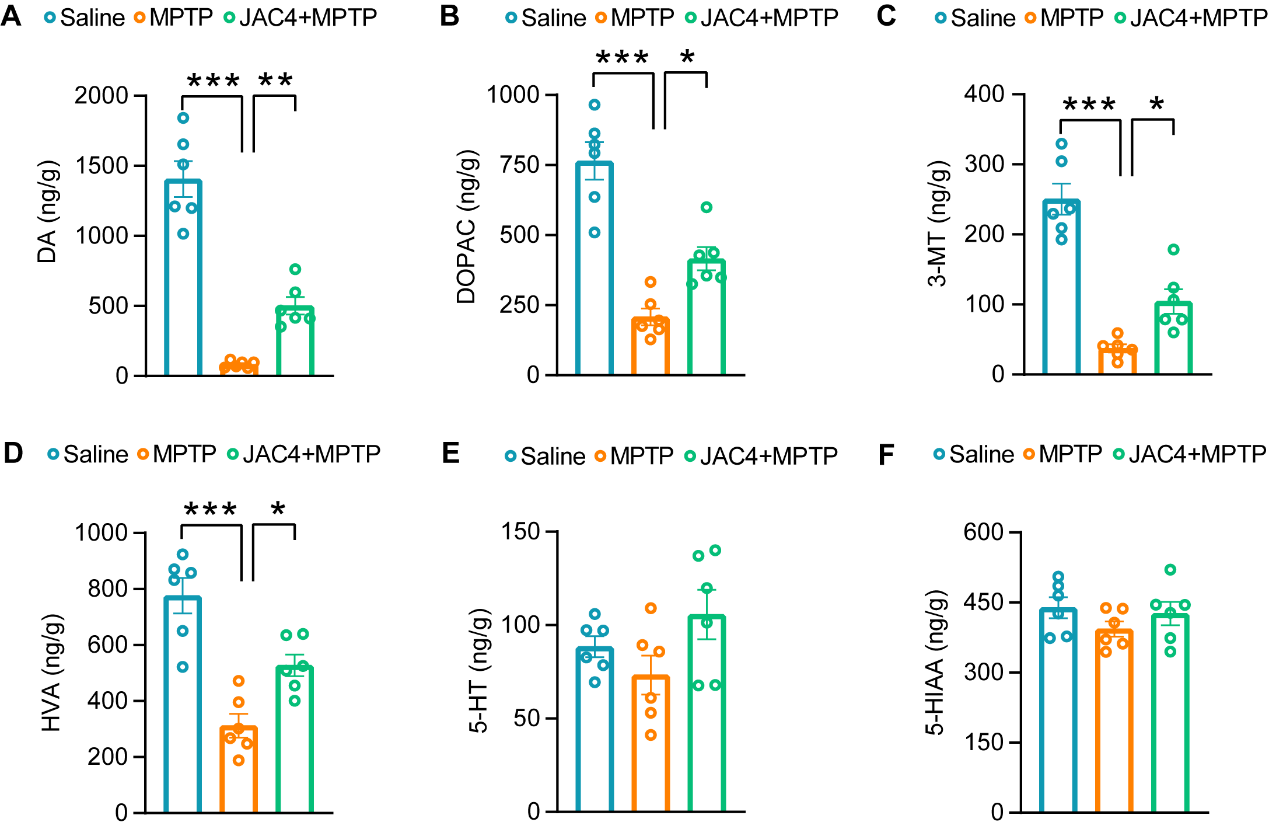


**Figure S6. JAC4 administration elevates the levels of dopamine and its metabolites in the striatum of MPTP-treated mice.**

**(A-D)** Measurement of dopamine and its metabolites levels in the striatum of mice treated with JAC4 (100 mg/kg) in the MPTP model. *p<0.05, **p<0.01, ***p<0.001 by one-way ANOVA with Tukey’s multiple comparisons test, n=6. **(E-F)** Determination of 5-HT and 5-HIAA levels in the striatum of mice treated with JAC4 (100 mg/kg) in the MPTP model. One-way ANOVA with Tukey’s multiple comparisons test, n=6.


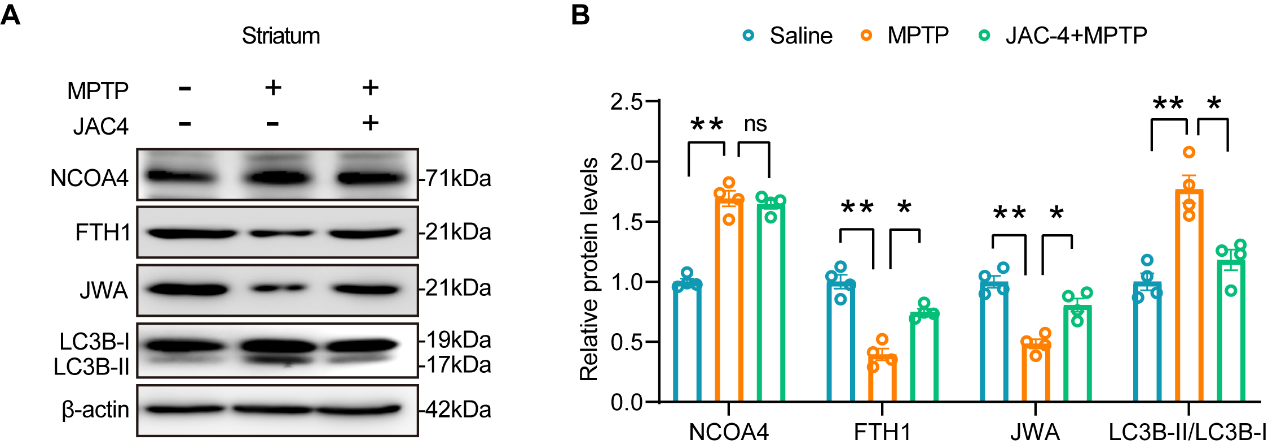


**Figure S7. Effects of JAC4 treatment on ferritinophagy in the striatum of PD mice.**

**(A-B)** Western blotting analysis and quantification of ferritinophagy-related proteins in the striatum of mice treated with JAC4 (100 mg/kg) in the MPTP model. *p<0.05, **p<0.01 by one-way ANOVA with Tukey’s multiple comparisons test, n=4, ns: no significance.


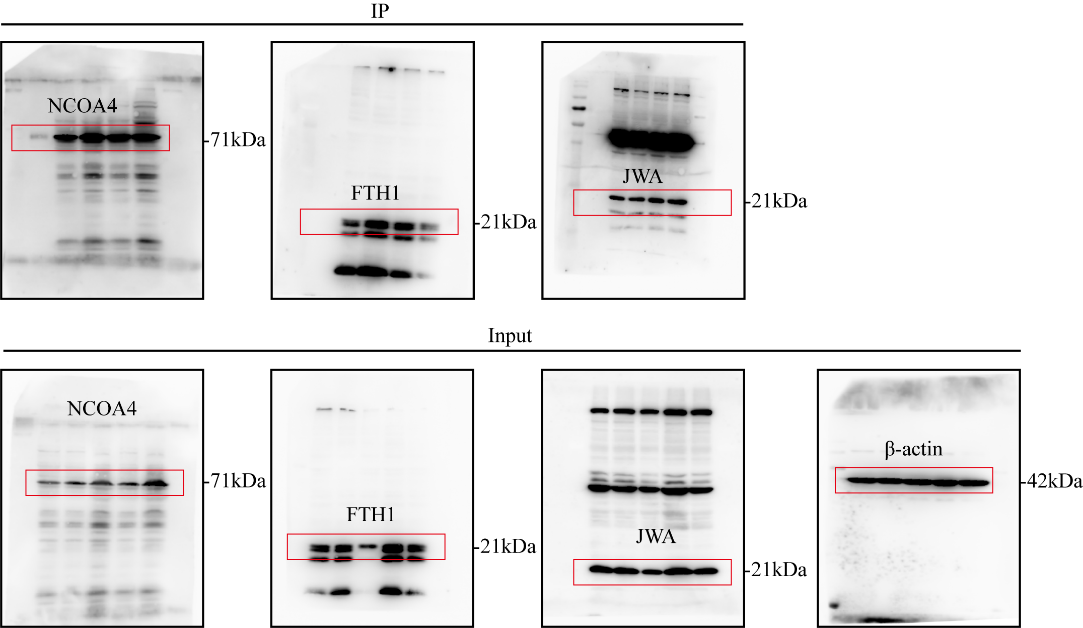


**Figure S8. The complete bands of Fig. 4G.**

**Supplementary Table 1. The main reagents and antibodies used in this study.**

| **Reagents & Antibodies** | **Source** | **Identifier** |
| --- | --- | --- |
| MPTP | Selleck | Cat# S4732 |
| MPP^+^ | Sigma-Aldrich | Cat# D048 |
| JAC4 | Prof. Jianwei Zhou’s laboratory | |
| Deferiprone | MedChemExpress | Cat# HY-B0568 |
| Erastin | MedChemExpress | Cat# HY-15763 |
| Ferrostatin-1 | MedChemExpress | Cat# HY-100579 |
| FerroOrange | DOJINDO | Cat# F374 |
| C11 BODIPY 581/591 | Invitrogen | Cat# D3861 |
| LysoTracker Deep Red | Invitrogen | Cat# L12492 |
| Hoechst 33342 | Invitrogen | Cat# H1399 |
| Anti-TH | Merck Millipore | Cat# MAB318 |
| Anti-TH | Merck Millipore | Cat# AB152 |
| Anti-ARL6IP5 (JWA) | Sigma-Aldrich | Cat# SAB1306837 |
| Anti-β-actin | Proteintech | Cat# 6609-1-Ig |
| Anti-NCOA4 (ARA70) | Santa Cruz Biotechnology | Cat# sc-373739 |
| Anti-FTH1 (Ferritin) | Abcam | Cat# ab75973 |
| Anti-LC3B | Cell Signaling Technology | Cat# 2775 |
| Anti-IRP1 | ABclonal | Cat# A4821 |
| Anti-IRP2 | ABclonal | Cat# A22283 |
| Annti-MAP2 | Proteintech | Cat# 67015-1-Ig |
| VeriBlot | Abcam | Cat# ab131366 |
| Alexa Fluor 555 donkey anti-rabbit secondary antibody | Invitrogen | Cat# A-31572 |
| Alexa Fluor 488 goat anti-mouse secondary antibody | Invitrogen | Cat# A55058 |
| Alexa Fluor 488 donkey anti-goat secondary antibody | Invitrogen | Cat# A11055 |
| Alexa Fluor 488 goat anti-rabbit secondary antibody | Invitrogen | Cat# A-11008 |
| HRP goat anti-mouse secondary antibody | Invitrogen | Cat# 31430 |
| HRP donkey anti-goat secondary antibody | Invitrogen | Cat# PA1-28664 |

**Supplementary Table 2. The primer sequences used in this study.**

| **Gene symbol** | **Primer sequences** |
| --- | --- |
| *PTGS2* | *PTGS2* (F): CTGGCGCTCAGCCATACAG  *PTGS2* (R): CGCACTTATACTGGTCAAATCCC |
| *ALOX15* | *ALOX15* (F): GGGCAAGGAGACAGAACTCAA  *ALOX15* (R): CAGCGGTAACAAGGGAACCT |
| *ACSL4* | *ACSL4* (F): CATCCCTGGAGCAGATACTCT  *ACSL4* (R): TCACTTAGGATTTCCCTGGTCC |
| *SLC7A11* | *SLC7A11* (F): TCCTGCTTTGGCTCCATGAACG  *SLC7A11* (R): AGAGGAGTGTGCTTGCGGACAT |
| *GPX4* | *GPX4* (F): ACAAGAACGGCTGCGTGGTGAA  *GPX4* (R): GCCACACACTTGTGGAGCTAGA |
| *FTH* | *FTH* (F): CCCCCATTTGTGTGACTTCAT  *FTH* (R): GCCCGAGGCTTAGCTTTCATT |
| *FTL* | *FTL* (F): CAGCCTGGTCAATTTGTACCT  *FTL* (R): GCCAATTCGCGGAAGAAGTG |
| *NCOA4* | *NCOA4* (F): ACAGTTGCATAAGCCGTCACC  *NCOA4* (R): TGAGCCTGCTGTTGAAGTGTC |
| *TFR* | *TFR* (F): ACCATTGTCATATACCCGGTTCA  *TFR* (R): CAATAGCCCAAGTAGCCAATCAT |
| *FPN1* | *FPN1* (F): CTACTTGGGGAGATCGGATGT  *FPN1* (R): CTGGGCCACTTTAAGTCTAGC |
| *DMT1* | *DMT1* (F): TGGAGATCATGGGGAGTCTG  *DMT1* (R): AAGAAAACCTGGTCCGGTGAA |
| *IREB2* | *IREB2* (F): TCGATGTATCTAAACTTGGCACC  *IREB2* (R): GCCATCACAATTTCGTACAGCAG |
| *GAPDH* | *GAPDH* (F): GCACCGTCAAGGCTGAGAAC  *GAPDH* (R): TGGTGAAGACGCCAGTGGA |
| *Jwa (Mus musculus)* | *Jwa* (F): CGGCATCACTCTTCCTTTGCTG  *Jwa* (R): CTTCCTGCTGTTCCAAGGCATC |
| *Gapdh (Mus musculus)* | *Gapdh* (F): CCTGGAGAAACCTGCCAAGTA  *Gapdh* (R): TCATACCAGGAAATGAGCTTGAC |
